# Perspective: Biomarkers of Aging in Human Nutrition Research—A Focus on Applications, Challenges, and Opportunities^☆^

**DOI:** 10.1016/j.advnut.2025.100486

**Published:** 2025-07-28

**Authors:** Keeva NM Loughlin, Pol Grootswagers, Guido Camps, Lisette CPGM de Groot

**Affiliations:** 1 Division of Human Nutrition and Health, Wageningen University, Wageningen, The Netherlands; 2 OnePlanet Research Center, Wageningen, The Netherlands

**Keywords:** human nutrition, longevity, biological aging, biomarkers of aging, aging clocks

## Abstract

Predictive algorithm-based biomarkers of aging (BoA), such as aging clocks, are increasingly applied within human nutrition research. Despite great promise of these BoA, validation efforts and guidelines for implementation are lagging behind the vast and growing number of available biomarkers, complicating their use and introducing variance across studies. Therefore, in the current perspective paper, we provide practical insights and an initial set of recommendations for consistent future implementation of BoA within nutrition research based on current knowledge, both on a general level and within different research scenarios. We critically reflect on existing observational and experimental nutrition research, and outline the potential application of BoA in identifying at-risk groups, exploring heterogeneity underlying aging and nutritional effects, and personalized approaches. This work aims to support nutritional researchers in making informed decisions on contextually appropriate biomarkers and provides directions for future nutritional research involving BoA, because, despite much needed advancements, we consider BoA exciting and promising tools in nutrition research.

**Statements of Significance**

To our knowledge, this is the first critical reflection on implementation of biomarkers of aging in human nutrition research, based on which we offer novel practical insight and actionable recommendations.

## Introduction

As the proportion of individuals aged over 65 continues to gradually increase, the impact of age-related disability and morbidity is rising [1,2]. Aging is an intricate, heterogenous process involving the time-dependent accumulation of damage from structural and functional alterations, causing a decline in function, higher vulnerability to disease, and eventually mortality [3,4]. Biological age (BA), rather than chronological age (CA), is thought to be better at capturing these deteriorative processes caused by aging [5,6]. Considering the long human lifespan, there is a need for reliable, quantifiable biomarkers of aging (BoA) that reflect biological aging and future health [7,8]. Such BoA are relevant for the identification and assessment of (dietary) longevity interventions within shorter timeframes (≤2 y), to improve knowledge on drivers and mechanisms of healthy aging.

**FIGURE 1:**
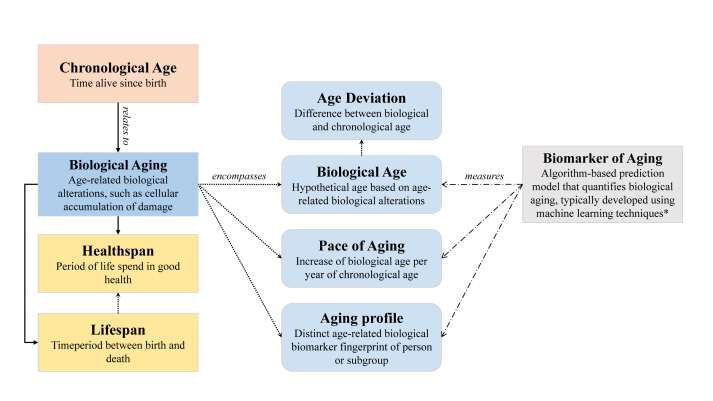
Conceptual framework of key terms discussed in the current perspective article. Solid arrows (─>) indicate “relates to,” dotted arrows (···>) “encompasses,” and the dashed-dotted arrows (─ ─>) “measures.” In part, definitions adapted from [10]. ∗Definition current article.

BoA range from conventional functional outcomes, such as gait speed and handgrip strength, to molecular markers, like omics, and comprise single or multivariate composite markers [9]. In the last decade, considerable progress has been made regarding the development of algorithm-based composite BoA, such as aging clocks, which are essentially prediction models that integrate various biological features associated with aging or health into one measure of biological aging, typically developed using machine learning (ML) techniques [[10], [11], [12]]. Compared with single BoA, generally composite BoA seem to better capture the complexity of aging, by containing less noise irrelevant to aging and better predicting age-related health outcomes [10,13,14]. Therefore, this article focuses on algorithm-based composite BoA, from now on referred to as BoA (Figure 1 for important concepts).

Given the clear link between nutrition and aging, BoA are increaslingly applied within human nutrition research. Individual variation in lifespan and longevity explained by genetics seems to decline throughout lifetime, emphasizing the pivotal role of environmental and lifestyle factors [[15], [16], [17]]. Prospective research has consistently shown that age-related morbidity and mortality are amenable to diet [18,19]. Among others, diet plays a role in healthy cognitive aging, muscle maintenance, bone health, and consequently quality of life [20]. Importantly, dietary strategies are usually inexpensive, unpatentable, and thus applicable across geographical and economical boundaries, thereby not leading to increased health disparities. Although it is generally known what constitutes a healthy diet, further specification and personalization could refine healthy aging guidelines. The significance of BoA within nutrition research not only lies in their ability to assess (effects on) aging in relatively short timeframes, but also, for ideal BoA that capture early signals of future age-related implications, the assessment of intervention in younger or preclinical populations [10,21]. Earlier intervention may contribute to the delay of onset or the prevention of age-related diseases, thereby extending the healthspan [20,22,23].

Despite the promise of these BoA in nutrition research, validation efforts and guidelines for implementation are lagging behind the vast and growing number of available markers, complicating use and introducing variance across studies. Therefore, there is a need to critically evaluate the predictive value, applicability, and considerations for implementation of existing BoA, to ultimately formulate guidelines for standardized use and future research directions. Lastly, beyond evaluating dietary longevity interventions in overall populations, BoA may serve as useful tools for other largely unexplored applications, like personalized nutritional strategies.

Considering the above, in this perspective paper, we aim to provide practical insights and an initial set of recommendations for future implementation of BoA within nutrition research. First, we briefly describe, on a general level, the development of BoA and related implications for implementation. Secondarily, we provide an extensive overview of the current applications of BoA in nutrition research, the challenges and limitations thereof, and subsequent actionable recommendations for future implementation. Furthermore, we present potential applications of BoA, and to conclude a perspective regarding future directions.

## Biomarkers of Aging

### Background on the development of BoA

**FIGURE 2:**
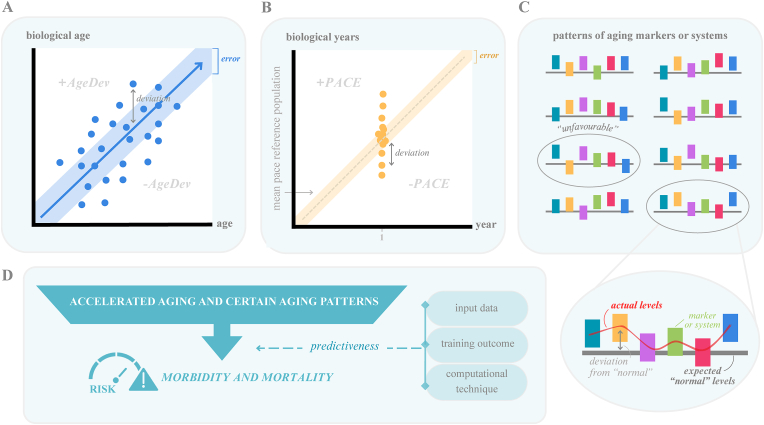
Three categories of composite algorithm-based biomarkers of aging. (A) Aging clocks predict an individual’s biological age (blue dots). AgeDev is the difference between biological and chronological age. (B) Pace of aging clocks measure the increase in biological years per chronological year (yellow dots). Pace of aging is the deviation from the reference mean. AgeDev and pace of aging can be positive/accelerated, well predicted (i.e., within error range) or negative/decelerated. (C) Aging patterns illustrate profiles of individual or systems of aging markers (colored boxes). Deviations between the actual measured levels (red line) and the expected normative levels given someone’s age (gray line), may be unfavorable for aging (circled in gray). (D) Accelerated aging and certain unfavorable patterns associate with morbidity and mortality risk. Biomarker predictiveness depends on development methodologies. AgeDev, age deviation.

Increased availability of high-throughput biological data through publicly available datasets and concurrent advancement of ML techniques have facilitated rapid development of BoA [11,24]. BoA are algorithm-driven models that provide information on biological aging and the cumulated risk of adverse health effects beyond CA. Although there are no standard guidelines for BoA development, typically model features are selected and corresponding weights are allocated based on associations between the input data and the outcome of interest, for example, CA [25], phenotype [26], or mortality [27]. Guidelines for criteria, terminology, characterization, and validation of BoA, as well as elaborate overviews of available BoA, have been presented elsewhere [[9], [10], [11], [12],^21^,^28^,^29^]. Among the numerous available BoA (*n* > 100), we have identified 3 main categories: *1*) aging clocks, *2*) pace of aging clocks, and *3*) aging patterns (Figure 2).

*Aging clocks* are the most common among the BoA currently addressed (our estimation is roughly 95%). Aging clocks reflect past aging up until the moment of measurement and are constructed using cross-sectional data from age-diverse samples. Well-known examples are the epigenetic Horvath [25], PhenoAge [26], and GrimAge [27] clocks. First, the generated algorithm predicts a composite score for BA. Subsequently, BA is typically regressed on CA to derive the relative AgeDev, being the residual of the regression. As such, the mean AgeDev of a given population, corresponding to the sum of residuals, amounts to zero. In select cases, an alternative method to derive AgeDev is subtracting CA from BA (correcting for CA), for example, Klemera-Doubal Method (KDM) and MetaboAge [30,31], wherein AgeDev is still based on the comparison between BA and CA, but not relative to the study population. Of note, AgeDev is also referred to as “Age Acceleration” or “Age Gap,” but AgeDev is the proposed standardized term [10]. For clocks trained on CA, positive or negative deviations exceeding the error in the age-predicting models, for example, mean absolute error (MAE), are generally considered meaningful accelerations or decelerations of aging, respectively [32]. Deviations that fall within the error range are regarded well predicted (i.e., BA and CA align) [32]. A positive or accelerated AgeDev has been related to an increased risk of adverse health outcomes, for example, functional decline, (time-to) morbidity, and mortality [26,27,33,34].

*Pace of aging clocks* reflects the speed of aging at the moment of measurement. So far, 2 such clocks have been constructed: DunedinPoAM, and its refined version DunedinPACE [8,35]. These markers have been derived from longitudinal data of age-matched individuals, yet can determine the pace of aging, defined as the increase in biological years per chronological year, from measurement at a single timepoint. Currently available pace of aging clocks have been scaled to the mean pace of the training dataset (i.e., ∼1), hence functioning as a reference [8,35]. As with aging clocks, deviations beyond error, above or below the mean, indicate aging at an accelerated or decelerated pace, respectively. Similarly, the accelerated pace of aging has been associated with adverse health outcomes [35,36].

*Aging patterns* describe distinct age-related biomarker profiles of individuals or subgroups. Currently, 2 patterns are available, each developed using distinct methodologies, having applied either cross-sectional or longitudinal profiling [37,38]. Patterns have different characteristics and may indicate differential underlying pathways of aging or dysregulation, for example, accelerated aging of certain organs or systems [37,38]. Within a pattern, certain markers or pathways can deviate from a normal aging trajectory, and as such may associate with unfavorable health outcomes [37,38]. Similar patterns are shared among individuals and can be clustered [37]. The interpretation of these patterns is somewhat more complicated compared with the aforementioned types of BoA, as the outcome is not a value for BA. However, patterns could provide more information on the underlying mechanisms of aging and intra- and interindividual differences therein.

### Development methodologies determine biomarker performance and applicability

#### Input data

BoA have been constructed utilizing various types of input data, such as omics, routine blood biochemistry, imaging, and functional traits. Currently, epigenetic markers based on DNA methylation patterns of specific sets of 5′-C-Phosphate-G3′ (CpG) sites are the most prevalent and extensively studied BoA, and have consequently shown notable advancements in predictive capacity [33,35]. Omics data can be useful for early detection of aging in presymptomatic populations, whereas imaging and functional testing rely on age-related structural changes or clinical manifestations, and therefore may provide greater value at later stages [9,21,26]. Data with direct links to biological processes, for example, proteomics or measures of clinical phenotype, provide more interpretable (mechanistic) insight than indirect measures, for example, epigenetics, and may better predict aging consequences [11,13,26]. Different data likely capture different aspects of aging, thus multiomics (or other mixed data) approaches might enhance predictiveness [11]. However, assessment can be demanding, require significant amounts of costly data, and may consequently not be practical [21,29]. Contrastingly, an advantage of routine blood-biochemistry data and certain functional tests is the lower cost of assessment and availability [12]. There are different sources from which data can be derived, such as blood aliquots, saliva, urine, tests, and scans. Blood-based markers, being an indirect measure, may poorly reflect processes in other specific tissues, such as the brain or skeletal muscle [13,39]. Certain molecular markers may provide insight into cell/tissue-specific aging [26], though accessibility of a given source can strongly impact widespread BoA applicability. Collection of tissue biopsies can be very invasive, and is in some cases only possible postmortem (e.g., brain tissue biopsy) [40], which does not align with the feasibility criteria for BoA [10], yet may be valuable in specific contexts. Once sourced, data can be obtained through different analysis techniques, such as various microarrays for the determination of DNA methylation, nuclear magnetic resonance (NMR) or mass spectroscopy for metabolomics, and processed using varying transformations. Each technique, with different coverage and resolution, produces different data [28]. Hence, it is important to carefully consider which type of source and analysis technique(s) will provide the appropriate data for a particular marker within the scope of a given study a priori.

#### Training outcome

The vast majority of BoA have been trained on CA, particularly nonepigenetic markers. Regarding aging clocks, the more accurate CA is predicted, that is, small error margin, the less actual biologically relevant signals the marker seems to contain, because BA becomes an approximation of CA [41]. Additionally, accuracy has been shown to further improve through training in larger samples, raising the question of what is noise and what is relevance [11,41]. A simulation study in epigenetic clocks also showed that with increasing accuracy of CA prediction, the more predictive accuracy seems to be driven by stochastic processes, that is, random methylation drift [42]. Such markers are likely less susceptible to change, for example, through nutritional intervention, and seem less predictive of age-related health outcomes [26,41,42]. The practical value of accurately predicting CA seems more applicable to forensic than longevity research. Besides accuracy and reliability, the associations with relevant health outcomes should be considered with regard to applicability [13]. The more recent clocks indicate an emerging trend toward other training outcomes, such as health-related factors (e.g., phenotype, mortality), pace of aging, and mechanistic information [26,27,35,43,44]. Accordingly, clocks have been divided into the so-called generations. The first generation comprises aging clocks trained on CA (e.g., Hannum, Horvath, MetaboAge), the second generation includes aging clocks trained on health-related factors (e.g., PhenoAge, GrimAge), and the third generation includes the pace of aging clocks (e.g., DunedinPACE) [45]. A potential next generation is emerging including, for example, causal aging clocks trained on causality to CA, distinguished by their distinct computational technique (i.e., CausAge, DamAge, and AdaptAge) [45]. Second- and third-generation clocks have been shown to outperform first-generation clocks in the prediction of age-related health outcomes [33,[45], [46], [47], [48]]. Supplemental Figure 1 shows how the focus in literature is shifting from first-generation clocks to second- and third-generation clocks over time. For BoA trained on outcomes less concrete than CA, such as phenotype, it may be more informative to judge applicability by predictive capacity, reliability, and responsiveness to intervention than accuracy. Lastly, a concern regarding mortality as a training outcome, though relevant to epidemiological research, is that it is not usually a main outcome in trials, restricting applicability and clinical relevance [10,28].

#### Computational technique

Without providing extensive elaboration, predominantly ML approaches are applied to construct BoA, ranging from penalized linear regression (Ridge, LASSO and Elastic net) to deep learning models (including neural networks). Detailed descriptions and comparisons of ML methods for prediction have been provided elsewhere, for example [11,[49], [50], [51]]. The (dis)advantages of these different computational techniques affect performance and utility. (Penalized) linear models are easier to interpret compared with more complex models, whereas deep learning is better suited to capture intricate nonlinear aging processes, as well as handle large, high-dimensional datasets and missing values [7,11]. Conversely, deep learning models are less interpretable (black-box nature) and suboptimal for small datasets [11]. Generalizability, and thereby applicability, among others, depends on how models handle issues like multicollinearity and overfitting, as well as on the independent training, test and validation sets used [13]. Note that model feature selection depends on the best combination of predictors in a given dataset, which may not necessarily be the most relevant to or informative of aging. After development, additional computational techniques can be employed to improve (technical) performance of BoA. Refined versions of several epigenetic clocks were built using principal component analysis to minimize technical noise, improving their test–retest reliability [52]. Similarly, other postdevelopment refinements or computational steps have led to the development of different versions of the same marker, for example, GrimAge2 [53].

## BoA in Nutritional Research

### Current applications in nutrition research

Thus far, application of BoA in nutrition research has been confined to the identification and assessment of dietary longevity interventions at population level. TABLE 1, TABLE 2 [26,27,[54], [55], [56], [57], [58], [59], [60], [61], [62], [63], [64], [65], [66], [67], [68], [69], [70], [71], [72], [73], [74], [75], [76], [77], [78], [79], [80], [81], [82], [83], [84], [85], [86], [87], [88], [89], [90], [91], [92], [93], [94], [95], [96], [97], [98], [99], [100], [101], [102], [103]] provide overviews of current evidence from observational and intervention studies that analyzed relationships between dietary intake and BoA, published before July 2024. Literature was searched nonsytematically using PubMed, Scopus, and Google Scholar employing terms such as “clock∗” (not circadian), “biological age,” “diet,” “nutrition,” and “lifestyle,” and by checking reference lists of identified studies. Nutrition was not always the primary focus of the original study designs. Our intention was not to conduct a systematic review on potential nutritional strategies for healthy aging but rather to provide a thorough overview of the applications in the field of nutrition and aging research.

**TABLE 1:** Current evidence from observational. studies on antiaging nutritional strategies.

Study	Population	Dietary assessment	Biomarkers of aging	Statistical method	Significant findings
Nutrients					
Phang et al., 2020 [54]	*N* = 169 Australian newborns aged around 38.7 ± 1.5 wk	Maternal dietary and supplemental nutrient intake from FFQ	HorvathDNAmAge^e^ (saliva)	Cross-sectional analysis with multivariable linear regression	HorvathAgeDev ↓ - Vitamin D supplementation HorvathAgeDev ↑ - Fat intake - Saturated fat intake - Monounsaturated fat intake - Palmitoleic acid intake - Oleic acid intake - Palmitic acid intake
Vetter et al., 2020 [55]	*N* = 1649 German young and older adults aged 28.9 ± 3.1 and 68.8 ± 3.7 y, respectively, from the Berlin Aging Study II^1^	Dietary and supplemental vitamin D from blood	7CpG-DNAmAge^e^	Cross-sectional analysis with multivariable linear regression	7CpG-DNAmAgeDev ↑ - Vitamin D status deficient vs. sufficient

Nwanaji-Enwerem et al., 2021 [56]	*N* = 715 American older community-dwelling men aged 72.6 ± 6.7 y from the VA Normative Aging Study	Dietary and supplemental one-carbon metabolites from blood	HorvathDNAmAge, GrimAge and PhenoAge^e^	Cross-sectional analysis with Bayesian kernel machine regression	PhenoAgeDev and GrimAgeDev ↓ - Vitamin B6 status PhenoAgeDev and GrimAgeDev ↑ - Folate status
Vetter et al., 2022 [57]	*N* = 1036 German older adults aged 68.3 ± 3.5 y from the GendAge study (follow-up of the Berlin Aging Study II)	Dietary and supplemental vitamin D from blood	7CpG-DNAmAge, HorvathDNAmAge, HannumDNAmAge, PhenoAge and GrimAge^e^	Cross-sectional and longitudinal (*n* = 126) analysis with multivariable linear regression	Cross-sectional 7CpG-DNAmAgeDev and GrimAgeDev↓ - Vitamin D status Longitudinal 7CpG-DNAmAgeDev ↓ - Vitamin D sufficiency at follow-up through supplementation in individuals who were deficient at baseline
Senior et al., 2022 [58]	*N* = 1560 Canadian community-dwelling older adults aged 76.8 ± 4.3 y from the Quebec NU-AGE study	Canadian Nutrient File based macro and micronutrients from 24 h recalls	BloodPhenoAge^b^ and KDM-Age^m^	Cross-sectional analysis with generalized additive models	PhenoAgeDev and KDM-AgeDev ↓ - High carbohydrate and lower to moderate protein intake
Gaskins et al., 2023 [59]	*N* = 61 American females undergoing ovarian stimulation aged 34.8 ± 5.7 y from the EARTH study	Supplemental folic acid intake from FFQ	CGmAge, HorvathDNAmAge and GrimAge^e^ (granulosa cells)	Cross-sectional analysis with multivariable linear regression	n.s.

Xing et al., 2023 [60]	*N* = 3193 American adults aged 47.6 ± 0.4 y from NHANES 2007–2010 and 2017–2018	Dietary flavonoid intake from 24 h recalls^2^	aKDM-Age, HeartAge, KidneyAge and LiverAge^m^	Cross-sectional analysis with multivariable linear regression	aKDM-AgeDev ↓ - Tertile 2 and 3 vs. 1 for total flavonoid, isoflavone, anthocyanidin, flavan-3-ol, flavanone, and flavone intake - Tertile 3 vs. 1 for flavonol intake HeartAgeDev ↓ - Tertile 2 and 3 vs. 1 for isoflavone and flavone intake - Tertile 3 vs. 1 for total flavonoid, anthocyanidin, and flavan-3-ol intake KidneyAgeDev ↓ - Tertile 2 and 3 vs. 1 for anthocyanidin intake - Tertile 3 vs. 1 for flavanone intake LiverAgeDev ↓ - Tertile 2 and 3 vs. 1 for total flavonoid, isoflavone, anthocyanidin, flavan-3-ol, flavanone, flavone, and flavonol intake
Ma et al., 2024 [61]	*N* = 4,692 American adults aged ∼49.4 ± 17.4 y from NHANES 2015–2018	Macro and micronutrients from 24 h recalls^2^	BloodPhenoAge^b^	Cross-sectional analysis with multivariable linear and restricted cubic spline regression	PhenoAgeDev↓ - Protein intake - Vitamin E intake - Vitamin A intake - Beta-carotene intake - Vitamin B1 intake - Vitamin B2 intake - Vitamin B6 intake - Vitamin K intake (nonlinear) - Phosphorus intake - Magnesium intake - Iron intake - Zinc intake - Copper intake - Potassium intake - Dietary fiber intake
Kawamura et al., 2024a [62]	*N* = 144 Japanese older men aged 68.0 ± 1.9 y from the WASEDA’S Health Study	Macro and micronutrients from BDHQ	HorvathDNAmAge, HannumDNAmAge, PhenoAge, GrimAge, and FitAge^e^	Cross-sectional analysis with partial correlation analysis and multivariable linear regression	Partial correlations HannumAgeDev ↓ - Iron intake - Copper intake - Manganese intake - Beta-carotene intake PhenoAgeDev ↓ - Vitamin C intake - Beta-carotene intake GrimAgeDev ↓ - Carbohydrate intake - Iron intake - Copper intake - Vitamin C intake - Beta-carotene intake FitAgeDev ↓ - Iron intake - Copper intake - Beta-carotene intake Multivariable PhenoAgeDev ↑ - Low beta-carotene intake GrimAgeDev ↑ - Low vitamin C intake
Zhu et al., 2024 [63]	*N* = 26,381 American adults aged 50.2 ± 17.9 y from NHANES 1999–2010 and 2015–2018	Macronutrients and subtypes thereof from 24 h recall(s)^2^	BloodPhenoAge^b^ and KDM-Age^m^	Cross-sectional analysis with multivariable linear regression	PhenoAgeDev and KDM-AgeDev ↓ - Dietary fiber intake - High-quality carbohydrate intake - (Plant) protein intake - (Omega-3) polyunsaturated fatty acid intake PhenoAgeDev and KDM-AgeDev ↑ - Low-quality carbohydrate intake - (Long-chain) SFA intake
Food groups					
Quach et al., 2017 [64]	*N* = 4173 North American postmenopausal females aged 64.0 ± 7.1 y from WHI and 402 Italian adults aged 71.0 ± 16.0 y from InCHIANTI^3^	Food groups from FFQ and dietary blood markers	HannumDNAmAge and HorvathDNAmAge^e^	Cross-sectional analysis with multivariable linear regression	HannumAgeDev ↓ - Fish - Carotenoid status HorvathAgeDev ↓ - Poultry
Levine et al., 2018 [26]	*N* = 4308 North American postmenopausal females aged 50–79 y from WHI^3^	Food groups and macronutrients from FFQ, as well as dietary blood markers	PhenoAge^e^	Cross-sectional analysis with biweight midcorrelation	PhenoAgeDev ↓ - Nuts - Carotenoid status PhenoAgeDev ↑ - Red meat
Lu et al., 2019 [27]	*N* = 4079 North American postmenopausal females aged around 64.0 ± 7.0 y from WHI^3^	Food groups and macronutrients from FFQ, as well as dietary blood markers	GrimAge^e^	Cross-sectional analysis with biweight midcorrelation	GrimAgeDev ↓ - Carbohydrates - Dairy - Whole grains - Fruit - Vegetables - Carotenoid status GrimAgeDev ↑ - Fat - Red meat
Wu et al., 2023 [65]	*N* = 421,764 adults of European ancestry from the UK Biobank (sample 1) and 34,710 from 28 different cohorts (sample 2)	Dried fruit intake from shortened touchscreen FFQ	GrimAge, HannumDNAmAge, HorvathDNAmAge, and PhenoAge^e^	Cross-sectional analysis with two-sample (multivariable) Mendelian randomization	PhenoAgeDev and GrimAgeDev ↓ - Dried fruit (raisins, dried plums, dried apricots)
Biemans et al., 2024 [66]	*N* = 3990 North American postmenopausal females aged 63.3 ± 7.1 y from WHI^3^	MyPyramid and item-level food groups from FFQ	PhenoAge^e^	Cross-sectional analysis with Copula Graphical Models	PhenoAgeDev ↓ - Peaches/nectarines/plums - Poultry - Nuts - Butter - Discretionary oil - Discretionary solid fat PhenoAgeDev ↑ - Eggs - Organ meat - Sausages - Cheese - Legumes - Starchy vegetables - Added sugar - Lunch meat - Fat added after cooking
Dietary patterns					
Dugué et al., 2018 [67]	*N* = 2818 Australian adults aged 59.0 ± 7.6 y from the MCCS	(a)Healthy Eating Index-2010, Mediterranean diet and components thereof from FFQ	HannumDNAmAge and HorvathDNAmAge^e^	Cross-sectional analysis with multivariable linear regression	HannumAgeDev ↓ - Fruit HorvathAgeDev ↑ - (red and processed) meat
Esposito et al., 2021 [68]	*N* = 4592 Italian adults aged 55.6 ± 11.7 y from the Moli-sani cohort	Polyphenol antioxidant content and total antioxidant capacity from FFQ	Gialluisi DNN-Age^b^	Cross-sectional analysis with multivariable linear regression	DNN-AgeDev ↓ - PAC-score
Kim et al., 2022a [69]	*N* = 1995 North American adults aged 67.0 ± 9.0 y from the FHS Offspring cohort	DASH diet, (a)Healthy Eating Index-2015, Mediterranean diet and components thereof from FFQ	DunedinPoam, GrimAge, and PhenoAge^e^	Cross-sectional analysis with linear mixed-effect regression	DunedinPoam ↓ - DASH - (a)HEI-2015 - Mediterranean diet - Nuts and legumes GrimAgeDev ↓ - DASH - (a)HEI-2015 - Mediterranean diet - Vegetables - Fruit - Nuts and legumes GrimAgeDev ↑ - Red and processed meat - Sodium PhenoAgeDev ↓ - DASH - aHEI-2015 - Mediterranean diet - Vegetables - Fruit - Nuts and legumes
Kresovich et al., 2022 [70]	*N* = 2694 non-Hispanic White American females aged 56.0 ± 9.0 y from the Sister Study	DASH diet, (a)Healthy Eating Index-2010; 2015 and Mediterranean diet from FFQ	GrimAge, HannumDNAmAge, HorvathDNAmAge, and PhenoAge^e^	Cross-sectional analysis with multivariable linear regression	GrimAgeDev and PhenoAgeDev ↓ - DASH - (a)HEI-2010 - (a)HEI-2015 - (a)Mediterranean diet HannumAgeDev ↓ - (a)HEI-2010
Esposito et al., 2022 [71]	*N* = 4510 Italian adults aged 55.6 ± 11.6 y from the Moli-sani cohort	Mediterranean diet, DASH diet, Paleolithic diet and Nordic Diet Index from FFQ	Gialluisi DNN-Age^b^	Cross-sectional analysis with multivariable linear regression	DNN-AgeDev ↓ - Mediterranean diet - DASH
Kim et al., 2022b [72]	*N* = 744 Black and White American adults aged 45.9 ± 3.5 y the from the CARDIA cohort	(a)Healthy Eating Index-2010 (average over time) from self-reported dietary history	GrimAge and PhenoAge^e^	Cross-sectional analysis with multivariable linear regression, quantile-based g-computation and Bayesian kernel machine regression	GrimAgeDev ↓ - (a)HEI-2010 Among lifestyle factors (a)HEI-2010 was the strongest negative relative contributor, but smoking ranked most important to AgeDev.
Hu et al., 2022 [73]	*N* = 16,681 American noninstitutionalized, nonpregnant females with cardiometabolic disease aged 53.1 ± 17.7 y from NHANES 1999–2010	Dietary Inflammatory Index and DASH diet from 24 h recall(s)^2^	BloodPhenoAge^b^ and KDM-Age^m^	Cross-sectional analysis with multivariable linear regression	PhenoAgeDev and KDM-AgeDev ↓ - DASH PhenoAgeDev and KDM-AgeDev ↑ - Tertile 2 and 3 vs. 1 for DII - DII
Thomas et al., 2023 [74]	*N* = 42,625 American adults aged 47.0 ± 16.7 y from NHANES 1999–2018	Mediterranean diet and Healthy Eating Index-2015 from 24 h recall(s)^2^	BloodPhenoAge^b^	Cross-sectional analysis with multivariable linear regression	PhenoAgeDev ↓ - Mediterranean diet - HEI-2015
Martínez et al., 2023 [75]	*N* = 4510 Italian adults aged 55.6 ± 11.6 y from the Moli-sani cohort	Energy adjusted-Dietary Inflammatory Index and Dietary Inflammation Score from FFQ	Gialluisi DNN-Age^b^	Cross-sectional analysis with multivariable linear regression	DNN-AgeDev ↑ - e-DII - DIS
Xie et al., 2023 [76]	*N* = 35,575 American adults aged 49.8 ± 18.2 y from NHANES 1999–2018^3^,^4^	Dietary Inflammatory Index from 24 h recall(s)^2^	BloodPhenoAge^b^ and KDM-Age^m^	Cross-sectional analysis with multivariable linear regression and smoothed curve fitting analysis	PhenoAgeDev ↑ - DII KDM-AgeDev ↑ - DII (nonlinear)
Zhang et al., 2023 [77]	*N* = 44,094 British adults aged 64.0 ± 8.0 y from the UK Biobank	Healthy diet (adequate consumption ≥ 4 out of 7 healthy food categories) from FFQ	ECG-Age^i^	Cross-sectional analysis with simple linear regression	ECG-AgeDev ↓ - Healthy diet
Wang et al., 2023 [78]	*N* = 10,191 Taiwanese adults aged 58.6 ± 6.5 y from the Taiwan MJ cohort	(overall, healthful, unhealthy) Plant-based Diet Index from FFQ	MD-Age^m^	Longitudinal analysis with multivariable multinomial logistics regression	MD-AgeDev↓(trajectory) - PDI - hPDI MD-AgeDev ↑ (trajectory) - uPDI
He et al., 2024 [79]	*N* = 25,305 American Adults 45.4 ± 16.1 y from NHANES 2001–2018	Composite Dietary Antioxidant Index from 24 h recall(s)^2^	BloodPhenoAge^b^	Cross-sectional analysis with multivariable linear regression	PhenoAgeDev ↓ - CDAI
Wang et al., 2024 [80]	*N* = 8839 American adults aged around 48.0 y from NHANES 2003–2014	Dietary Inflammatory Index and Dietary Oxidative Balance Score from 24 h recalls^2^	BloodPhenoAge^b^ and KDM-Age^m^	Cross-sectional analysis with binomial logistic, multivariate linear and restricted cubic spline regression	PhenoAgeDev ↓ - Tertile 2 and 3 vs. 1 for DOBS KDM-AgeDev ↓ - Tertile 3 vs. 1 for DOBS (nonlinear) PhenoAgeDev ↑ - Tertile 2 and 3 vs. 1 for DII (nonlinear) - High DII + low DOBS vs. low DII + high DOBS KDM-AgeDev ↑ - Tertile 2 and 3 vs. 1 for DII - High DII + low DOBS vs. low DII + high DOBS
Li et al., 2024 [81]	*N* = 1041 Australian adults aged 57.4 ± 7.9 y from the MCCS	(a)Healthy Eating Index-2010, Mediterranean diet and Dietary Inflammatory Index from FFQ	GrimAge, PhenoAge, PCGrimAge, PCPhenoAge, and DunedinPACE^e^	Cross-sectional and longitudinal analysis with multivariable linear regression	Cross-sectional^5^ GrimAgeDev ↓ - (a)HEI-2010 - Mediterranean diet PCGrimAgeDev & DunedinPACE ↓ - (a)HEI-2010 GrimAgeDev ↑ - DII Longitudinal GrimAgeDev ↓ - (a)HEI-2010 PhenoAgeDev and DunedinPACE ↓ - (a)HEI-2010 - Mediterranean diet GrimAgeDev, PhenoAgeDev and DunedinPACE ↑ - DII
Thomas et al., 2024 [82]	*N* = 1644 North American older adults aged 69.6 ± 6.9 y from the FHS Offspring cohort	MIND diet (average over time) from FFQ	DunedinPACE^e^	Cross-sectional analysis with multivariable linear regression	DunedinPACE ↓ - MIND
Kawamura et al., 2024b [83]	*N* = 144 Japanese older men aged 68.0 ± 1.9 y from the WASEDA’S Health Study	Healthy Japanese and Western dietary pattern from BDHC	HorvathDNAmAge, HannumDNAmAge, BioAge4HAStatic, SkinBloodAge, PhenoAge, GrimAge, and FitAge^e^	Cross-sectional analysis with partial correlation analysis and multivariable linear regression	n.s.
Liu et al., 2024 [84]	*N* = 88,039 British adults aged 57 (IQR 46–62) y from the UK Biobank	Dietary Diversity Score and components thereof from 24 h recalls	BloodPhenoAge^b^ and KDM-Age^m^	Cross-sectional analysis with generalized linear and restricted cubic spline regression	PhenoAgeDev and KDM-AgeDev ↓ - High vs. low DDS - DDS (nonlinear) - Grains - Vegetables - Fruit KDM-AgeDev ↓ - Milk products
Chiu et al., 2024 [85]	*N* = 342 American Black and White females aged 39.1 ± 1.1 y from the NHLBI-GHS Social Epigenomics Program	(a)Mediterranean diet, (a)Healthy Eating Index-2010, Epigenetic Nutrient Index and added sugar intake from 3-day food record	GrimAge2^e^ (saliva)	Cross-sectional analysis with multivariable linear regression	GrimAge2Dev ↓ - (a)Mediterranean diet - (a)HEI-2010 - ENI GrimAge2Dev ↑ - Added sugar
Kuiper et al., 2024 [86]	*N* = 24,332 European middle-aged to older adults from the DCS, RS and UK Biobank^6^	Healthy dietary adherence from FFQ^6^	MetaboAge, MetaboHealth	Cross-sectional analysis with linear regression and mixed-effects models	MetaboHealth ↓ - Healthy diet
	*N* = 14,969 British adults aged 57.7 ± 7.4 y from the UK Biobank	Healthy diet (adequate consumption ≥ 4 out of 7 healthy food categories) from FFQ	MetaboAge, MetaboHealth	Longitudinal analysis with linear mixed-effects models.	MetaboAgeDev ↑ - Healthy diet MetaboHealth ↓ - Healthy diet
Reynolds et al., 2024 [87]	*N* = 4500 North American postmenopausal females aged 65.0 ± 7.0 y from WHI^3^	Healthy Eating Index-2015, DASH diet and (a)Mediterranean diet from FFQ	HannumDNAmAge, HorvathDNAmAge, PhenoAge, GrimAge, and DunedinPACE^e^	Cross-sectional analysis with linear mixed models.	HorvathAgeDev ↓ - HEI-2015 PhenoAgeDev, GrimAgeDev, and DunedinPACE ↓ - HEI-2015 - DASH - (a)Mediterranean diet

When possible mean age ± SD or median age (IQR) provided. Biomarkers of aging measured in blood unless stated otherwise (^b^, blood biochemistry; ^e^, epigenomics; ^I^, imaging or scans; ^m^, multiomics or mixed data). ↑ indicates positively associated with positive AgeDev, whereas ↓ indicates negatively associated with positive AgeDev. A positive AgeDev relates to the risk of adverse health outcomes, for example, morbidity or mortality. The table exclusively shows results for the overall population, not subanalyses, from the most fully adjusted models available in that study. The studies included focused on composite biomarkers that were trained and tested in 2 different samples or datasets and published in peer-reviewed articles, dietary intake from diet or supplements, assessment methods (predominantly) reflecting intake of >1 d. Studies on beverages, lacking a clear description of dietary assessment or other important methodology and that misreported results were excluded. Studies published online before July 2024 were included. Note the literature search was not performed systematically; thus, it is possible studies are missing from the overview.

Abbreviations: (a), alternate; AgeDev, age deviation (between biological and chronological age, the lower the better); BDHQ, Brief-type self-administered Dietary History Questionnaire; CARDIA, Coronary Artery Risk Development in Young Adults; DASH, Dietary Approaches to Stop Hypertension; DCS, Doetichem Cohort Study; DDS, Dietary Diversity Score; DII, Dietary Inflammatory Index; DIS, Dietary Inflammation Score; DNAm, DNA methylation; DNN, deep neural network; DOBS, Dietary Oxidative Balance Score; EARTH, Environment And Reproductive healTH; ENI, Epigenetic Nutrient Index; FFQ, Food Frequency Questionnaire; FHS, Framingham Heart Study; (a)HEI, (alternate) Healthy Eating Index; KDM, Klemera-Doubal Method; MCCS, Melbourne Collaborative Cohort Study; MD, multidimensional; MIND, Mediterranean-DASH Intervention for Neurodegenerative Delay; NHLBI-GHS, National Heart, Lung, and Blood Institute Growth and Health Study; n.s., not significant; PAC, polyphenol antioxidant content; PC, principal component; RS, Rotterdam Study; WHI, Women’s Health Initiative.

1 Ratio young to old was about 1:4.

2 NHANES 1999–2002 1 24 h recall, 2003 ≥ 2 24 h recalls.

3 WHI data used from ancillary studies BA23 and EMPC (AS315).

4 It was not possible to determine PhenoAge in all participants; the population size for this analysis was *n* = 26,433.

5 Cross-sectional analysis performed in baseline and follow-up data. The current table only shows associations that were significant at both timepoints.

6 DCS was a Dutch population (*n* = 4446) aged 55.6 ± 9.9 (used modified Dutch Healthy Diet 2015 Index to study diet), RS a Dutch population (*n* = 4719) aged 71.0 ± 8.1 (used adherence to Dutch Health Council guidelines), and UK Biobank a British population (*n* = 14,969) aged 57.7 ± 7.4 y (used healthy diet score).

**TABLE 2:** Current evidence from intervention studies on antiaging nutritional strategies.

Study	Population	Duration	R	Nutritional intervention	Control intervention	Biomarkers of aging	Outcome
Supplements							
Obeid et al., 2018 [88]	*N* = 63 German older adults aged 68.4 ± 10.1 y	1 y	✓	Daily supplementation with 0.5 mg vitamin B9, 50 mg vitamin B6, 0.5 mg vitamin B12, 1200 IU vitamin D3, 800 mg Ca-carbonate (*n* = 32)	Daily supplementation with 1200 IU vitamin D3 and 800 mg Ca-carbonate (*n* = 31)	aWeidnerDNAmAge^eαβ^	aWeidnerAgeDev ↑ - Intervention vs. control [OR = 5.26 (95% CI: 1.51, 18.28); *P* = 0.009]
Sae-Lee et al., 2018 [89]	*N* = 44 Dutch older adults with mildly elevated homocysteine levels aged 70.8 ± 2.9 y from the B-PROOF study	2 y	×	Daily supplementation with 400 μg folic acid and 500 μg vitamin B12	No control	HorvathDNAmAge^eαβ^	n.s.
	*N* = 13 Dutch nonobese, healthy smoking men with mean age 48 y (range 30–58 y)	8 wk	×	Daily supplementation with 200 μg monomeric and oligomeric flavanols	No control	HorvathDNAmAge^eαβ^	n.s.
Chen et al., 2019 [90]	*N* = 51 overweight or obese African Americans with suboptimal vitamin D status aged 26.1 ± 9.3 y	16 wk	✓	Daily supplementation with vitamin D3 Arm 1: ∼600 IU (*n* = 12) Arm 2: ∼2000 IU (*n* = 15) Arm 3: ∼4000 IU (*n* = 12)	Placebo (*n* = 11)	HorvathDNAmAge and HannumDNAmAge^eαβ^	HorvathAgeDev ↓ - 2000 and 4000 vitamin D3 (arm 2 and 3) vs. placebo and 600 IU vitamin D3 (arm 1) [*β* = –1.49 (95% CI: –2.78, –0.21); *P* = 0.023] - 4000 IU vitamin D3 (arm 3) vs. placebo [*β* = –1.85 (95% CI: –3.66, –0.03); *P* = 0.046] HannumAgeDev ↓ - 2000 IU vitamin D3 (arm 2) vs. placebo [β = –1.90 (95% CI: –3.75, –0.05); *P* = 0.044]
Jenkins et al., 2022 [91]	*N* = 1470 American predominantly White men aged 32.6 ± 5.9 y from the FAZST study	6 mo	✓	Daily supplementation with 5 mg folic acid and 30 mg zinc (*n* = 713)	Placebo (*n* = 757)	Germ-LineDNAmAge^eα^ (sperm)	n.s.
Dioum et al., 2022 [92]	*N* = 191 Canadian adults with moderate-to-elevated LDL-cholesterol levels aged 47.6 ± 11.4	4 wk	✓	Daily supplementation with 3 g oat β-glucan (*n* = 96)	Rice powder control (*n* = 95)	iAge^pα^	n.s.
McGee et al., 2024 [93]	*N* = 80 British healthy, predominantly White older adults aged 71.9 ± 6.2 y	12 wk	×	Daily supplementation with 20 μg vitamin D3, 50 mg vitamin B3, 85 mg vitamin C, 250 mg EPA and DHA, 10 mg hydroxytyrosol, 30 mg resveratrol, and 3.2 mg astaxanthin	No control	HorvathDNAmAge, HannumDNAmAge, and mean Epi-Age^eβ^	n.s.
Michels and Binder, 2024 [94]	*N* = 16 German healthy unfortified men aged 39.2 ± 11.0 y	8 wk	✓	Daily supplementation with folic acid arm 1: 400 μg (*n* = 9) arm 2: 800 μg (*n* = 7)	No control	HorvathDNAmAge, HannumDNAmAge, PhenoAge, GrimAge, GrimAge2 and DunedinPACE^e^	n.s.
Dietary patterns							
Belsky et al., 2017 [95]	*N* = 220 American nonobese young to middle-aged adults aged 38.0 ± 7.0 y from the CALERIE 2 study	2 y	✓	25% caloric restriction from baseline intake (*n* = 145)^1^	Ad libitum habitual diet (*n* = 75)	KDM-Age^mα^	KDM-AgeDev ↓ - Intervention vs. control [β = –0.60 (95% CI: −0.99, −0.21); *P* = 0.003].
Waziry et al., 2023 [96]						PCGrimAge, PCPhenoAge, DunedinPACE, HorvathDNAmAge, PCHorvathDNAmAge, HannumDNAmAge, PCHannumDNAmAge, Skin&BloodAge and PCSkin&BloodAge^eα^	DunedinPACE ↓ Intervention vs. control [intent-to-Treat 12mo *d* = –0.26 (95% CI: –0.42, –0.10); *P* = 0.001; 24 mo d = –0.24 (95% CI: –0.40, –0.07); *P* = 0.005; treatment on the treated 12 mo *d* = –0.36 (95%CI: –0.59, –0.13); *P* < 0.001; 24mo n.s.]
Gensous et al., 2020 [97]	*N* = 60 Italian elderly aged 72.2 ± 3.8 y and 60 Polish elderly aged 71.1 ± 4.1 y from the NU-AGE study	1 y	×	Mediterranean-style diet	No control	HorvathDNAmAge and HannumDNAmAge^eα^	n.s.
Multimodal lifestyle							
Petersen et al., 2021 [98]	*N* = 16 rural American obese White, non-Hispanic older adults aged 73 ± 5.7 y	12 wk	×	Diet (focus on caloric restriction, fiber and vitamin D) – exercise weight loss intervention	No control	HannumDNAmAge, HorvathDNAmAge and PhenoAge^e^	n.s.
Yaskolka Meir et al., 2021 [99]	*N* = 120 predominantly male adults with abdominal obesity or dyslipidemia aged 60.3 ± 7.5 y from the CENTRAL MRI study	18 mo	✓	Arm 1: hypocaloric low fat diet (*n* = 30) Arm 2: Mediterranean low carbohydrate diet (*n* = 30) Arm 3: hypocaloric low fat diet and physical activity (*n* = 30) Arm 2: Mediterranean low carbohydrate diet and physical activity (*n* = 30)	No control	LiDNAmAge^eα^	n.s.
Fitzgerald et al., 2021 [100]	N = 43 healthy American predominantly White men aged ∼59.7 ± 6.5 y	8 wk	✓	DNA methylation diet^2^, guidance on exercise, sleep and relaxation, and supplemental probiotics and phytonutrients (*n* = 21)	No intervention (*n* = 22)	HorvathDNAmAge^eβ^	HorvathAgeDev ↓ - Intervention vs. control (Δ= –3.23, *P* = 0.018)
Fiorito et al., 2021 [101]	*N* = 219 postmenopausal females aged ∼59.0 ± 5.2 y from the DAMA study	2 y	✓	Arm 1: Mediterranean-style diet (*n* = 56) Arm 2: physical activity (*n* = 55) Arm 3: Mediterranean-style diet and physical activity (*n* = 52)	General advice on diet and physical activity (*n* = 56)	GrimAge^eα^	GrimAgeDev ↓ - Dietary (arms 1 and 3) interventions vs. physical activity (arm 2) intervention and control [Δ = –0.42 (95% CI: –0.83, –0.01); *P* = 0.05]
Fitzgerald et al., 2023 [102]	*N* = 6 females aged 57.8 ± 6.8 y	8 wk	×	DNA methylation diet,^2^ guidance on exercise, sleep and relaxation, and supplemental probiotics and phytonutrients	No control	HorvathDNAmAge^eβ^	HorvathgeDev ↓ - Intervention (Δ = –4.60, *P* = 0.39)
Yaskolka Meir et al., 2023 [103]	*N* = 256 Israeli predominantly male adults with abdominal obesity or dyslipidemia aged 51.3 ± 10.6 y from the DIRECT PLUS study	18 mo	✓	Arm 1: caloric-restricted Mediterranean diet and physical activity (*n* = 81) Arm 2: caloric-restricted polyphenol-rich, low-red/processed meat Green-Mediterranean diet and physical activity (*n* = 87)	Healthy dietary and physical activity guidelines (*n* = 88)	HorvathDNAmAge, LiDNAmAge, HannumDNAmAge, Skin&BloodAge, PhenoAge, PCGrimAge, DunedinPACE^eα^	n.s.

When possible, mean age ± SD or range provided. Biomarkers of aging measured in blood unless stated otherwise (^e^, epigenomics; ^p^, proteomics; ^m^, multiomics or mixed data). ↑ indicates the intervention increased AgeDev, whereas ↓ indicates the intervention decreased AgeDev. A positive AgeDev relates to the risk of adverse health outcomes, for example, morbidity or mortality. The table exclusively shows results for the overall population, not subanalyses, from the most fully adjusted models available in that study. The outcomes reflect the difference in biological aging between endline and baseline on intervention, and if possible in contrast to the control group. The studies included focused on composite biomarkers that were trained and tested in 2 different samples or datasets and published in peer-reviewed articles, and dietary intake from diet or supplements. Studies on beverages and those lacking a clear description of dietary intervention or other important methodology were excluded. Studies published online before July 2024 were included. Note the literature search was not performed systematically; thus, it is possible studies are missing from the overview.

Abbreviations: AgeDev, age deviation (between biological and chronological age); DNAm, DNA methylation; PC, principal component; R, randomized (✓ yes; **×** no).

^α^Biomarker of aging not primary study outcome (either secondary or post hoc).

^β^Not corrected for cell counts.

1 Average caloric restriction adherence was 11.9% rather than aimed 25%.

2 The DNA methylation diet was plant-centered and intermittent fasting, rich in nutrients involved in one-carbon metabolism and restricted simple carbohydrates.

### Observational studies

In our literature search, we identified a considerable number (*n* = 36) of observational studies on the relation between nutrition and various BoA, among which the vast majority were cross-sectional and used epigenetic aging clocks. Despite design-related limitations, such as residual confounding, and that most effect sizes were small, several recurring trends of potentially promising dietary components or patterns are observed. Some dietary components have been more frequently studied than others, due to hypothesis-based analyses, and therefore recur more often, whereas other components remain largely unexplored. Generally, associations appear stronger for dietary patterns than single nutrients or food groups, as would be expected because dietary components are consumed in combination and have intricate interactive effects [104]. Additionally, associations seem generally stronger for markers trained on outcomes other than CA. Data were mainly sourced from large publicly available datasets, like the NHANES and the Women’s Health Initiative, but also smaller studies wherein sample sizes were often limited (5 of the 36 studies had *N* < 350) [54,59,62,83,85]. Study populations consisted of middle-aged to older adults, predominantly from European ancestry.

Among nutrients, several studies found an inverse relationship (implying higher intake relates to lower AgeDev) between carotenoids, such as beta-carotene, and AgeDev, as measured by different aging clocks [26,27,62,61,64]. Although explored to a lesser extent, similarly negative associations with AgeDev were found for iron [61,62], copper [61,62], vitamin B6 [56,61], and vitamin D [54,55,57]. For macronutrients, associations with AgeDev seemed to rely on subtypes and sources. AgeDev was observed to be inversely associated with high-quality carbohydrates (e.g., fiber) and certain fatty acids, while positively associated with low-quality carbohydrates (e.g., added sugar) and other fatty acids [54,61,63,66,85]. Findings reported for protein intake were somewhat contradictory, with inverse relationships with AgeDev being observed for both (plant) protein and restricted protein intake [58,61,63]. Previous research has suggested that potential negative effects may relate to animal-based proteins [105], which is consistent with the positive associations found between AgeDev and red and processed meats [26,27,66,67], cheese and eggs [66], but not the inverse associations with poultry and fish [64,66]. Findings for legumes appear mixed, though when studied as food group combined with nuts, inverse relationships with AgeDev are observed [66,69], possibly attributable to nuts. Overall vegetable and fruit intake was not or weakly negatively associated with AgeDev [26,27,64,67,69,84]. At item level, relationships among dried fruit, peaches, starchy vegetables, and AgeDev were found [65,66]. Thus, it is crucial to, where possible, investigate subcategories of nutrients and food groups, as effects may differ across these categories.

As for dietary patterns, several studies focused on plant-rich diets, using scores such as the Mediterranean diet score, the Dietary Approaches to Stop Hypertension (DASH) score and Healthy Eating Index. These diets all prioritize intake of vegetables, fruits, wholegrains, and legumes, restrict sugar and sweets, yet somewhat differ in recommendations for fat, meat, dairy, and sodium [[106], [107], [108], [109]]. Despite differences, similar negative trends were shown in the associations with aging. Though effect sizes varied considerably among studies, which is likely partially attributed to discrepancies in BoA and methodologies used. Overall, a trend is observed linking dietary inflammatory capacity with aging, which is not unexpected considering low-grade chronic inflammation is a hallmark of aging [110]. The Mediterranean (MED) and in lesser degree DASH diet have demonstrated anti-inflammatory potential in previous research [111,112]. Moreover, as presented in Table 1 [26,27,[54], [55], [56], [57], [58], [59], [60], [61], [62], [63], [64], [65], [66], [67], [68], [69], [70], [71], [72], [73], [74], [75], [76], [77], [78], [79], [80], [81], [82], [83], [84], [85], [86], [87]], weak-to-moderate associations were found among the dietary inflammatory index, dietary antioxidant content, and biological aging [68,73,75,76,79,80,81].

### Experimental studies

Several nutritional intervention studies have reported effects on BoA. These studies predominantly included BoA as secondary or post hoc outcomes, and were therefore initially designed for other purposes, or were pilot studies. Consequently, studies varied considerably in population size and study duration. Several studies were limited by sample size (*n* ≤ 16 per arm) [89,90,94,98,100]. Most supplementation interventions focused on nutrients involved in one-carbon metabolism. Remaining studies mainly investigated MED-style diets or dietary restriction strategies, like caloric restriction (CR) and intermittent fasting (IF). Most interventions solely included epigenetic clocks. Among those that measured blood-based methylation, approximately half did not perform the important correction for circulating white blood cell proportions [113]. Correcting for cell counts is especially crucial in longitudinal studies, also for nonblood tissues with cellular heterogeneity, as methylation patterns differ among cell types, and composition can differ across measurements and the aging trajectory [113]. The majority of trials solely included CA-trained aging clocks, yet the more recent studies applied next-generation BoA. About half of the studies assessed only one BoA and/or did not include a (appropriate) control group for comparison. Given the above, results should be considered carefully.

Despite the direct link between one-carbon metabolism and methylation, results of the supplementation trials were largely insignificant [88,89,91,94]. One randomized controlled trial (RCT) found that after 1-year vitamin B supplementation, the intervention group had a significantly higher WeidnerAgeDev than the control group (Odds Ratio = 5.26) [88]. The effect may partly relate to the concentration of vitamin B6 provided (50 mg), which was above the tolerable upper limit (12.5 mg) [114]. However, limitations compromise the reliability of results: AgeDev was included as a binary outcome, and the intervention group seemed to have more participants with a positive AgeDev to begin with, whereas effects were only considered at endline. Furthermore, similar to observational findings, decelerating effects were found after vitamin D supplementation in individuals with low vitamin D status [90], yet may merely reflect a change in cell composition or noise [52]. None of the supplementation trials accounted for dietary intake in statistical analyses, which is especially important for those without an appropriate control.

CR interventions showed mixed effects. KDM-AgeDev and DunedinPACE more slowly increased after 2 y of 11.9% CR, compared with a habitual diet (KDM *β* = −0.60; DunedinPACE *d* = −0.24), with a dose–response effect for DunedinPACE [95,96]. Though, effects were insignificant for several (*n* = 8) other markers [96]. In older obese populations, different multimodal interventions, including CR, did not find significant effects [98,99,103]. Among these was a small 12-wk uncontrolled study [98], but also 2 larger 18-mo studies wherein effects were compared with different reference interventions [99,103]. Considering the average amount of weight loss (<4% body weight) together with mean baseline BMI (>30 kg/m^2^), compliance to the CR regime seems limited. Similarly, the trial with 11.9% CR initially aimed for 25% [95,96]. Thus, despite the promising effects of CR in animal studies [105,115], adherence to proper CR proves challenging for humans and therefore real-life implementation may not be feasible. Fasting mimicking diets (FMD) may be a better approach; however, these remain to be studied in relation to BoA. Another multimodal pilot study, including an IF plant-focused diet, was found to decelerate HorvathAgeDev by 3.23 y compared with control after 8 wk [100], with similar results in a uncontrolled replication case study [102]. Despite these promising findings, owing to small sample size, not using the principle component clock version and lacking confounder adjustment, these should be interpreted with extreme caution. As applies to other multimodal interventions, considering the intervention comprised several lifestyle components, it is unknown which effects can be attributed to diet. Furthermore, 1 study on a MED-style diet found effects on GrimAgeDev (Δ = –0.42) [101]. Comparably, in another study, higher green-MED diet adherence was associated with lower AgeDev from 2 of 7 measures at endline (LiAge  *β* = −0.41, *P* = 0.004; HannumAge *β* = −0.38, *P* = 0.038 per SD) [103]. As the baseline Food Frequency Questionnaire did not assess all required components, change in adherence over time could not be determined. Lastly, an uncontrolled study did not find an effect of a 1-y MED-style diet in the overall population, but did find a significant reduction in HorvathAgeDev in Polish females only (*n* = 36) [97]. In conclusion, although observational data indicate a potential benefit of consuming a more plant-rich diet, as of yet supporting intervention-based evidence is limited. There is a need for well-designed intervention studies with aging as primary outcome, employing next-generation BoA. Several upcoming interventions have been preregistered, focusing on nutritional supplementation (NCT06666660, NCT06613542), IF (NCT05678426), and other strategies (NCT06440681).

### Challenges and limitations

#### BoA in general

First and foremost, the greatest challenge of using BoA is that their true meaning and predictive value remain somewhat ambiguous, limiting their present clinical usefulness. It is only recently that some standard guidelines for the validation of BoA were proposed, and up to date validation attempts have been minimal, due to persisting challenges [28]. It should be noted that some markers have been more extensively tested than others, for example, PhenoAge and GrimAge [28]. BoA remain proxies of biological aging with varying signal-to-noise ratios [52]. It still has to be evidenced how short-term changes in BoA translate into long-term health outcomes [21]. Related to this, it is yet to be determined what is considered a relevant change beyond significance for any BoA, for example, minimal clinically important difference. Most BoA have been constructed using cross-sectional data, and therefore mainly rely on associations rather than causal links with age-related outcomes, introducing potential bias [28,116]. Additionally, epigenetic clocks are currently the most implemented BoA, whereas the direction of causality among methylation and aging remains uncertain [110]. Longitudinal studies are required to improve our understanding of causality within the aging trajectory and how changes in aging are sustained. The recent introduction of causal-inference methods, like Mendelian randomization, into the BoA development process additionally offers promising mechanistic insight [43]. The current availability of longitudinal (multi) omics data is limited, especially at the same timepoints as nutrition data. Furthermore, non-White/European individuals are highly underrepresented in present data, and concurrently in data used for the development and validation of BoA [28]. Collective efforts among (interdisciplinary) research teams and institutes should be directed toward setting up new and enhanced data sharing of existing studies, preferably with measurements across several time points collecting multimodal data within the same individuals, including ethnically diverse populations [12,28].

As mentioned, often AgeDev is considered the residual of the regression of BA on CA. Therefore, AgeDev is relative to the sample mean of the specific population in question. Consequently, when there is a large discrepancy between BA and CA, for example, due to poor health, AgeDev will still be relative to that population, even though the population is shifted as a whole. This is not necessarily a problem, especially if differences between multiple timepoints within persons are compared. However, for cross-population comparison of results, we argue that it is important to report mean values for both CA and BA in the baseline characteristics table, which is frequently not the case, for example [70,81].

#### BoA in nutritional research

A significant challenge in nutrition research is limited study comparability, restricting evaluation of potential antiaging strategies. Studies varied regarding choice of BoA, methods employed for determination of BoA and dietary assessment, as well as how results are analyzed and reported.

The selection of BoA used in nutritional research is inconsistent. The lack of validation efforts and the overwhelming number of available markers complicate decision-making. Additionally, there are no standard guidelines on how to implement these markers, and as to which markers may be of interest within a certain context. We aim to provide insights to clarify these aspects in the present and subsequent sections. First, it is important to substantiate the choice for certain BoA, which is often unclear. BoA should not be applied indiscriminately, merely because data are available, without taking into account important considerations and limitations. Furthermore, biological aging is likely too complex to be captured by a single marker; therefore, studies should include multiple *n* ≥ 2 (complementary) markers to provide more complete information on different aspects of aging [7,11,12]. Yet, several nutritional studies included only one. Note, some studies in TABLE 1, TABLE 2 [26,27,[54], [55], [56], [57], [58], [59], [60], [61], [62], [63], [64], [65], [66], [67], [68], [69], [70], [71], [72], [73], [74], [75], [76], [77], [78], [79], [80], [81], [82], [83], [84], [85], [86], [87], [88], [89], [90], [91], [92], [93], [94], [95], [96], [97], [98], [99], [100], [101], [102], [103]] may have included other outcomes that were not shown, because these were not composite BoA. Besides, most studies exclusively applied epigenetic markers, whereas studying other or combining multiple data types may allow for more comprehensive phenotyping [11]. So far, BoA based on different omics have shown limited correlation, including metabolomic and epigenetic clocks, elucidating their dual assessment may be complementary [51,[117], [118], [119]]. To date, only 1 study investigated metabolomic clocks in relation to nutrition [86], highlighting opportunities for future research, as integration of metabolomics may provide a valuable mechanistic link between aging and nutrition. Lastly, combining several unrelated BoA into one measure does not guarantee enhanced predictive performance [7], and may complicate interpretation and comparison of results (e.g., meanEpiAge, combining first and second-generation clocks [93]).

Variance is also introduced by methodological diversity in determination of BoA and dietary intake. As previously stated, equivalent data for BoA can be obtained using varied (nonstandardized) approaches, for which future recommendations are provided elsewhere [28]. Similarly, dietary assessment methods, food group classifications, and dietary scores varied across studies. Characteristics and (dis)advantages of each dietary assessment method have been addressed in detail previously [120]. Beyond heavily relying on self-report methods, the reliability of available nutrition data can occasionally be questioned, for example, some datasets only have data available from a single 24-h recall or multiple single recalls months apart, which may inadequately capture day-to-day variation [120]. Hence, ensuring data quality and use of validated dietary assessment methods is crucial.

Different approaches were used to analyze and report data. Studies corrected for a (subjective) preselection of potential confounders, if correcting for confounders at all. Besides cell counts, it is recommended to at least (correctly) adjust for other known confounders like CA [121]. In select cases, methods used for the determination of AgeDev varied despite using the same BoA, for example, using “crude” BA rather than usual regression [98], or regression rather than usual subtraction [122] (excluded from Table 1 [26,27,[54], [55], [56], [57], [58], [59], [60], [61], [62], [63], [64], [65], [66], [67], [68], [69], [70], [71], [72], [73], [74], [75], [76], [77], [78], [79], [80], [81], [82], [83], [84], [85], [86], [87]]). AgeDev and dietary adherence scores were assessed as either continuous or categorical. We would recommend using continuous outcomes, to avoid loss of information and potential misclassification, for example, due to model error, AgeDev>0 does not necessarily imply accelerated aging. Furthermore, studies varied in metrics used to report findings, despite applying the same analysis method (e.g., *β*/unit or standardized β/SD from linear regression). Lastly, interpretation may be complicated when several markers are tested and results differ, for example [96,103]. Similarly, results from a single BoA may not extend to unmeasured others. Interpretation is highly dependent on research context and BoA-panel; thus, concrete standardized advice is not applicable. Some hypothetical situations and corresponding interpretations are provided in Supplemental Material 1. Naturally, it is misleading to conclude an overall effect if for instance only 2 of 8 outcomes show significant effects. It is important to decide beforehand what an effect on each BoA entails within a given study, and what defines a successful (intervention) effect. We strongly recommend preregistering future studies, wherein the to-be-included BoA-panel and interpretation criteria are stated, for transparency and to mitigate potential publication bias. Altogether, there is a need for increased consistency among studies to enable comparison, to strengthen the evidence base for nutritional longevity strategies. Recommendations to improve consistency are provided in Table 3 [28,121,123,124].

**TABLE 3:** Proposed guidelines for the consistent implementation of BoA.

Stage	Proposed guidelines^1^
1. Biomarker selection	- Use multiple *n* ≥ 2 (complementary) biomarkers, selected according to the research aim and comparable previous studies, and substantiate choices made. No formal maximum is set, provided markers are complementary and of (added) relevance. Complementarity of biomarkers can be evaluated through, for instance, the degree of correlation between them, their training outcomes, input data, and predictiveness of age-related health outcomes. - Focus on biomarkers trained on relevant physiological or other age-related outcomes, shown predictive of prospective health beyond chronological age and mortality. - Use the most recently updated or refined version of a biomarker (e.g., GrimAge2).
2. Data processing and analysis	- If available, use standard packages to determine biomarkers [28] (e.g., MiMIR [123] or BioAge [124]). If not, use the code provided in the original reference or related repository. - Correct for chronological age and other important known confounders, such as cell counts, dietary components (e.g., energy intake, nutrient(s) related to nutrient and outcome of interest, food group(s) related to food group and outcome of interest) and other lifestyle or environmental factors, if not controlled for within study design. For guidance on how to correct for chronological age, refer to [121]. Correcting for cell counts is especially critical within longitudinal studies. - Include biological aging and dietary intake metrics, such as AgeDev, as continuous outcomes.
3. Reporting of findings	- Report mean values for both chronological and biological age in the baseline characteristics when working with relative AgeDev from aging clocks. - Where possible, use standardized outcome measures for reporting results (e.g., *β* per SD). - Establish and preregister criteria for a successful antiaging strategy a priori, for instance, 2 of 3 of biomarkers show significant association or response, and draw conclusions accordingly.

Abbreviations: AgeDev, age deviation (between biological and chronological age).

1 On refinement of biomarkers of aging, more specific guidelines for standardized use can be formulated.

Finally, most implemented (cross-sectional) statistical analysis methods are inherently subject to limitations regarding handling interrelated nutrition data, that is, linear models and correlation analyses. These methods do not account for indirect effects between variables, possibly overlooking more complex multivariate interactions [125]. Besides, relationships between aging and diet may be nonlinear, as seen in, for example [61,80], whereas linearity is often assumed. Future studies focusing on nutrients (including supplements) or foods should at least adjust for important dietary components. Overall, exploring more powerful statistical tools, such as network analyses, for both data-driven and hypothesis-driven analyses would be valuable. To conclude, despite the great promise of BoA, there are several challenges and limitations that impact the quality of current evidence and need to be considered on implementation in future nutrition research.

#### Implementation recommendations within different nutrition research scenarios

Although it is premature to provide definitive guidelines on BoA for specific situations, we propose some directions and general recommendations for future implementation within different research scenarios. Table 3 [28,121,123,124] applies across all scenarios. Considerations related to study populations have been incorporated in Supplemental Material 1 as part of interpretation. An overview of BoA considered promising for nutrition research is provided in Table 4 [6,26,27,30,35,37,38,43,44,52,53,[126], [127], [128], [129], [130], [131], [132]], of which the selection process decision flowchart is depicted in Supplemental Figure 2. Among these, currently the most promising markers seem PCPhenoAge, GrimAge (PC or second version), and DunedinPACE, yet that may change as novel markers continue to emerge. Simultaneous assessment of these 3 measures may be of added predictive value, as they seem complementary. Whereas pace of aging clocks reflects current aging, aging clocks inform on BA over lifetime [10]. Correspondingly, although GrimAge and DunedinPACE are more strongly related (than with PhenoAge), there still seems to be added predictive value [35]. We would generally recommend using aging clocks, such as PhenoAge and GrimAge, as primary endpoint when the interest is past exposure, and pace of aging clocks, such as DunedinPACE, when the interest is current exposure, which we anticipate to adapt more rapidly than age accumulated over lifetime.

**TABLE 4:** Suggested biomarkers of aging currently relevant to nutrition research.

Biomarker (type)	Description	Measurement^1^	Trained on	Associated with^2^	Relevance	Implementation
Epigenomics						
PCPhenoAge [52] (aging clock)	Original [26]: two-step process: 1) identified 9 clinical blood markers and chronological age through Cox penalized regression in *n* = 9926 NHANES-III participants aged >20 y, tested in NHANES-IV; 2) identified 513 CpG sites through elastic net regression on blood methylation data from *n* = 456 InCHIANTI participants aged 21–91 y. PC: refined original model through principal component analysis using data from several population-based cohorts.	Measured from whole blood using Illumina BeadChip 450K or EPIC ($$$)	Phenotypic age	Mortality, cardiovascular disease, coronary heart disease, congestive heart failure.	- Higher test–retest reliability than original and requires smaller sample requirements for detecting effect - Outperforms first-generation epigenetic clocks - May capture early aging signals	Software package: PC-Clocks (https://github.com/MorganLevineLab/PC-Clocks) Outcome: AgeDev from the regression of BA on CA in population for analysis per timepoint
PCGrimAge [52] (aging clock)	Original [27]: two-step process: 1) Identified methylation-based surrogates for plasma protein variables and smoking pack-years through elastic net regression on blood methylation data from *n* = 2356 participants of the Framingham Heart Study Offspring Cohort aged around 66–67 y. 2) Identified 1030 CpG sites through elastic net Cox regression of time-to-death on methylation-based surrogates from step 1, age and sex. PC: refined original model through principal component analysis using data from several population-based cohorts.	Measured from whole blood using Illumina BeadChip 450K or EPIC ($$$)	All-cause mortality	Mortality, cardiovascular disease, coronary heart disease, congestive heart failure	- Slightly higher test–retest reliability than original and requires smaller sample requirements for detecting effect - Original outperforms previous epigenetic clocks (incl. original PhenoAge) and (already) has high test–retest reliability - No need to transfer data from local working environment (unlike GrimAge2) - May capture early aging signals	Software package: PC-Clocks (https://github.com/MorganLevineLab/PC-Clocks) Outcome: AgeDev from the regression of BA on CA in population for analysis per timepoint
GrimAge2 [53] (aging clock)	Original [27]: two-step process: 1) Identified methylation-based surrogates for plasma protein variables and smoking pack-years through elastic net regression on blood methylation data from *n* = 2356 participants of the Framingham Heart Study Offspring Cohort aged around 66–67 y. 2) Identified 1030 CpG sites through elastic net Cox regression of time-to-death on methylation-based surrogates from step 1, age and sex. Version 2: from the original 1030 CpG sites, identified methylation-based surrogates for 2 more plasma proteins through elastic net regression. Then, repeated step 2 from original model.	Measured from whole blood or saliva using Illumina BeadChip 450K or EPIC ($$$)	All-cause mortality	Time-to-death, time-to-coronary heart disease, time-to-congestive heart failure, time-to-cancer, comorbidity count, type 2 diabetes, hypertension, physical function level.	- Outperforms original - Original outperforms previous epigenetic clocks (incl. original PhenoAge) and has high test–retest reliability - May capture early aging signals	Software package: Online calculator (https://dnamage.clockfoundation.org/) Outcome: AgeDev from the regression of BA on CA in population for analysis per timepoint
DNAmFitAge [126] (aging clock)	Two-step process: 1) Identified 627 CpG sites as blood methylation-based surrogates for fitness parameters through LASSO penalized regression in participants from different datasets. 2) Constructed FitAge through KDM using the methylation-based fitness surrogates from step 1 and GrimAge.	Measured from whole blood using Illumina BeadChip 450K or EPIC ($$$)	Fitness parameters and chronological age	Time-to-death, time-to-coronary heart disease, hypertension.	- Potential utility in multimodal interventions with physical activity - May capture early aging signals	Software package: DNAmFitAge (https://github.com/kristenmcgreevy/DNAmFitAge) Outcome: AgeDev from the regression of BA on CA in population for analysis per timepoint
DunedinPACE [35] (pace of aging clock)	Two-step process: 1) Identified rate of longitudinal changes in organ-system biomarkers through linear mixed-effects modeling in *n* = 1037 age-matched Dunedin Study Birth Cohort participants, from which age-dependent annual changes were calculated to derive the pace of aging. 2) Identified 173 CpG sites through elastic net regression on blood methylation data as surrogate for pace of aging.	Measured from whole blood using Illumina BeadChip 450K or EPIC ($$$)^3^	Pace of aging	Mortality, physical and cognitive functioning, self-rated health, chronic disease, time-to-cardiovascular disease, time-to-stroke or transient ischemic attack.	- High test–retest reliability - Measures (20-y) longitudinal change from single timepoint - Outperforms previous epigenetic clocks (incl. original PhenoAge) similar to GrimAge, yet with added value - May capture early aging signals	Software package: DunedinPACE (https://github.com/danbelsky/DunedinPACE) Outcome: Pace of aging directly from PACEprojector() function
CausAge, DamAge, AdaptAge [43] (aging clock)	Identified CpG sites potentially causal to aging traits, separating damaging and protective changes, through epigenome-wide Mendelian randomization in data from several European cohorts, to then construct 3 clocks through elastic net regression in blood methylation data from *n* = 2664 individuals.	Measured from whole blood using Illumina BeadChip 450K ($$$)	Chronological age	Mortality, atherosclerosis, cancer prognosis,^4^ hypertensive heart disease,^4^ progeroid syndrome.^4^	- Includes putative causal CpGs offering mechanistic insight - Distinguishes between damage and protection - May capture effects short intentions and early aging signals	Software package: Methods described and model weights provided in supplement; for analysis in python bio-learn (https://bio-learn.github.io/) Outcome: AgeDev from the regression of BA on CA in population for analysis per timepoint
Metabolomics						
MetaboHealth [127] (aging clock)^5^	Identified 14 biomarkers related to mortality through Cox proportional hazard modeling in blood metabolite data from *n* = 44,168 participants aged 18–109 from 12 cohorts.	Measured from EDTA plasma or serum using Nightingale Health 1H-NMR-Metabolomics ($)	All-cause mortality risk	All-cause mortality, cause-specific mortality (cancer, cardiovascular, nonlocalized infections, other).^6^	- Outperformed conventional mortality risk factors - Cheap (yet, minimum batch requirement) - Link metabolites and nutrition	Software package: MiMIR (https://github.com/DanieleBizzarri/MiMIR) Outcome: Risk score from sum of transformed and weighted features
MetaboAgeMort [44] (aging clock)	Two-step process: 1) Identified mortality-associated metabolic markers through multivariable Cox regression, with further feature reduction to 35 markers and chronological age through LASSO Cox regression in 239,291 UK Biobank participants aged 58.3 (IQR: 50.6–63.7) 2) Constructed model through Gompertz proportional hazards regression.	Measured from EDTA plasma using Nightingale Health 1H-NMR-Metabolomics ($)	All-cause mortality	All-cause mortality, cause-specific mortality (cancer, cardiovascular disease, respiratory disease, digestive disease, neurodegenerative disease, other), range of morbidities (e.g., liver disease, type 2 diabetes, chronic kidney disease, respiratory disease).	- Outperformed conventional mortality risk factors and likely MetaboHealth (but no standard package) - Seems to capture signal beyond MetaboHealth - Cheap (yet, minimum batch requirement) - Link metabolites and nutrition	Software package: Other than description of methods, not (clearly) reported Outcome: AgeDev from the regression of BA on CA in population for analysis per timepoint
Proteomics						
Proteomic organ-specific clocks [128] (aging clock)	Identified organ-enriched proteins and trained organ aging models through (bootstrap aggregated) LASSO regression in *n* = 1398 Knight-ADRC participants aged 27–104, tested in 4 other cohorts.	Measured from plasma using SomaLogic SomaScan assay ($$$)	Chronological age (cognition models also global clinical dementia rating)	Different organ ages show different associations with mortality, morbidity (e.g., congestive heart failure, Alzheimer’s disease) and other health outcomes.	- Mechanistic insight - Some models more promising than others and some may add value to Alzheimer Disease biomarkers	Software package: organage (https://github.com/hamiltonoh/organage) Outcome: AgeDev from subtracting lowess curve (for BA versus CA) from BA
Multiomics						
IMM-AGE [129] (aging clock)^7^	Two-step process: 1) Identified 18 longitudinally dynamic age-dependent cell-subsets though different analysis methods, and trained model through principal component analysis and the diffusion maps algorithm in *n* = 135 participants, of which *n* = 63 aged 20–31 y and *n* = 72 aged 60–96 y. 2) Identified candidate gene set of which expression related to cellular IMM-AGE score and chronological age through linear regression, refined with Pearson correlation.	Measured from whole blood using Affyme-trix GeneChips (transcriptomics) ($$$)	Immune cell dynamics and chronological age	All-cause mortality, cardiovascular disease, cytokine response.	- Added value over conventional risk factors and more significant than Horvath clock in mortality prediction - (9 y) longitudinal profiling - Link inflammation, aging and nutrition	Software package: Code available on request Outcome: AgeDev from the regression of age score (based on gene set enrichment) on CA in population for analysis
Ageotypes [38] (aging pattern)	Identified 4 major ageotypes related to aging through grouping on pathway-enrichment analysis (Qiagen Ingenuity Pathway Analysis) on age-related markers from different omics found through Spearman correlation and rank-based linear regression in longitudinal data from 43 individuals aged 29–75 y.	Measured from plasma using LC-MS (metabolomics) and SWATH-MS (proteomics), from PBMCs using NGS (transcriptomics), from serum using targeted 62-plex Luminex assay (cytokines), from stool and nasal swab using NGS (microbial profiling) ($$$)	Chronological age	May be related to alterations in organ or system-specific function, but not tested.	- Comprehensive (2–3 y) longitudinal phenotyping - Mechanistic insight - Information on systems and individual level - May be sensitive to dietary changes - Concept may be more relevant than precise measure	Software package: Other than description of methods and some supplementary information, not (clearly) reported Outcome: Pattern in AgeDevs per ageotype system from the sum of regression coefficients
Mixed-data approaches						
Levine’s KDM-Age [6] (aging clock)	Identified age-related biomarkers through Pearson correlation and then applied KDM in data from *n* = 9389 NHANES-III participants aged 30–75 y.	Measured from serum, lung function, and blood pressure using different (laboratory) procedures^6^ ($-$$)	Chronological age	Mortality	- Relatively cheap - Adjustable according to data availability (also used to develop other clocks [126,130]) - KDM outperformed principal component analysis and multiple linear regression in mortality prediction	Software package: BioAge (https://github.com/dayoonkwon/BioAge) Outcome: AgeDev from subtracting CA from BA
Organ-system clocks [130,131] (aging clock)	Nie [130]: Identified 10 organs and systems from age-related features and trained models through KDM in *n* = 4066 Chinese Longitudinal Healthy Longevity Survey participants aged 20–45 y. Tian [131]: Identified 12 organ-specific phenotypes from age-related features and trained models through support vector machines in *n* = 143,423 UK Biobank participants aged 39–73 y, and (smaller) subsets aged 45–83 y.	Measured from physical and clinical assessments, several biospecimens (e.g., blood aliquots, stool, urine) and imaging using different (laboratory) procedures^6^ ($$$)	Chronological age	Different organ ages show different associations with mortality, morbidity, and other health outcomes.	- Mechanistic insight - Some associations between organ-specific age and dietary factors - Concept may be more relevant than precise measures	Software package: For [130], code provided (https://github.com/Detao-Zhang/bioage_Rscripts/tree/v1). For [131], some code for core analysis provided (https://github.com/yetianmed/BioAge) Outcome: In both cases, AgeDev from subtracting CA from BA, which for [131] is corrected by regression of this difference on CA in reference
Blood biochemistry						
Signatures of aging [37] (aging pattern)	Identified 26 signatures of aging (clusters) from 19 blood biomarkers that change with age through agglomerative cluster analysis in 4704 Long Life Family Study participants aged 30–110 y.	Measured from serum using different laboratory procedures^6^ ($$)	Chronological age	Different signatures show different associations with physiological markers (e.g., gait speed, FEV1), mortality, and morbidity (e.g., diabetes, cardiovascular disease).	- Relatively cheap - Mechanistic insight - Information on systems and subgroup level - Concept may be more relevant than precise measure	Software package: Other than description of methods and data for Bayesian classifier in supplement, not (clearly) reported Outcome: Cluster assigned to each individual
BloodPhenoAge [26] (aging clock)	Identified 9 clinical blood biomarkers and chronological age through Cox penalized regression in *n* = 9926 participants from NHANES-III aged >20 y, tested in NHANES-IV (step 1 of original PhenoAge)	Measured from whole blood and serum using different laboratory procedures^6^ ($-$$)	Phenotypic age	All-cause mortality, disease-specific mortality (age-related, CVD, cancer, Alzheimer’s, diabetes, chronic lower respiratory disease), comorbidity count, physical functioning.	- Relatively cheap - Better risk predictor than DNA methylation version of PhenoAge (closer proximity to risk outcomes) - Adjustable according to data availability	Software package: BioAge (https://github.com/dayoonkwon/BioAge) Outcome: AgeDev from subtracting CA from BA
Bortz Blood clock [132] (aging clock)	Identified 25 blood biomarkers through elastic net penalized Cox proportional hazard modeling in *n* = 306,116 UK Biobank participants.	Measured from whole blood and serum using different laboratory procedures^8^ ($-$$)	Survival time	All-cause mortality	- Outperformed Blood-PhenoAge in mortality prediction - Value imputation in case of certain unmeasured markers does not seem to substantially reduce accuracy	Software package: Bloodmarker_BA_estimation (https://github.com/bortzjd/bloodmarker_BA_estimation) Outcome: AgeDev from additive difference BA and CA, adjusted for sex, with features mean-centered.

Age provided if information available.

Abbreviations: 1H-NMR, proton nuclear magnetic resonance; incl., including; KDM, Klemera-Doubal method [30]; LASSO, least absolute shrinkage and selection operator; LC-MS, (untargeted) liquid chromatography–mass spectrometry; MGS, metagenomic sequencing; NGS, next-generation sequencing; PBMCs, peripheral blood mononuclear cells; SWATH-MS, sequential window acquisition of all theoretical fragment ion spectra mass spectrometry; WGS, whole-genome sequencing.

1 ($/$$/$$$) reflect prices per sample or measurement <€35/€35–150/>€150, respectively.

2 Only associations included from original references, not further (comparative) analyses.

3 Although developed in longitudinal data, DunedinPACE can be determined from single-timepoint blood measurement.

4 Not associated with all 3 CausAge, DamAge, and AdaptAge.

5 Not officially an aging clock because it does not provide biological age as outcome, but rather provides a risk score for mortality.

6 Associations with cause-specific mortality were assessed for the individual biomarkers included in MetaboHealth, and associations varied among them.

7 Exception to aging clocks because developed with longitudinal data, but output is biological age (based on place on axis) and not pace of aging.

8 NHANES-III procedures can be found on the cdc.gov website; UK Biobank procedures can be found on their website; Link to Long Life Family Study procedures seems to be broken.

#### Cross-sectional studies

Cross-sectional studies often explore associations between dietary components and biological aging. We posit that BoA should be selected according to the dietary assessment method applied. In case of methods that reflect long-term, past intake [120], we would recommend aging clocks as the main outcome, particularly when accounting for additional past behaviors or exposures. Whereas regarding methods that reflect short-term, current intake [120], we would recommend the pace of aging clocks. To determine baseline BA, aging clocks should be included as secondary outcome(s) [10].

#### Observational and experimental longitudinal studies

Observational longitudinal studies often involve long durations and intervals between measurements (several years); therefore, recommendations align with those for cross-sectional studies. Though it is more critical to ensure inclusion of BoA with high test–retest reliability to prevent false positive or negative findings due to technical noise [52]. Ideally, within longitudinal research, focus should be placed on surrogate endpoints, that is, markers that change in response to intervention and are able to predict (and alter) future health, yet currently no BoA has been validated as such [10]. In experimental studies, where current exposure is assessed, pace of aging is considered the preferred main outcome. A key consideration in longitudinal studies is duration. Robust short-term RCTs including BoA to inform on minimal durations are scarce, also for lifestyle factors other than nutrition. 16 and 8 wk were found sufficient to alter first-generation epigenetic clocks on nutritional supplementation and a multimodal intervention, respectively [90,100,102]. Yet, results may have been contaminated by cell composition and noise. Previous studies on profiles and single markers have similarly shown short durations (≤12 wk) suffice to detect alterations [[117], [118], [119],[133], [134], [135]], though this may not extend to BoA, as concurrent modification of several features is required for effect, prediction introduces a degree of uncertainty, and training outcomes often reflect the long-term, like 5- or 10-y mortality risk. Duration also depends on expected intervention strength, for example, relatively modest change in observational studies or single-nutrient interventions, compared with experimental or multimodal interventions, respectively. Although these considerations complicate advising on study duration, we propose provisional minimal durations of 16-wk for single-nutrient or food interventions in those with suboptimal levels or health, whereas probably 6-mo would be better in healthy populations, and 12-wk for whole diet and multimodal interventions. These are rough estimations based on limited available data and should be confirmed in future studies informing on responsiveness of BoA. Note, research on sustained change would involve longer follow-up durations, for example, assessing stability of methylation across type-specific cell divisions [136,137]. We expect roughly 1.5–2 y to be sufficient for assessment of BoA based on functional or structural changes [131,[138], [139], [140], [141]], with deviations depending on intervention strength, functional domain or structure type, and population characteristics, like age and health status. Several structural BoA determine BrainAge (*n* = ∼15), previous nutritional studies on (non-BoA) cognitive outcomes suggest the relevance of such relatively long durations, and the variability involved [142,143]. Responsiveness and reversibility of functional and structural BoA remain to be assessed.

#### Personalized approaches

Reliable and viable individual-level (*n* = 1) BoA do not exist as of yet, and therefore existing markers should not be used as such. Alongside systemic markers, mechanistically informed markers, organ-specific markers and aging patterns can provide information on individual differences and allow for analyses among subgroups or systems within individuals, which is the highest degree of personalization that we would recommend. In the potential applications section, personalized approaches are further discussed.

#### Limited budget

In the context of a limited budget, observational data can be obtained from (free) publicly available databases, and experimental data from markers based on routine blood-biochemistry and/or functional tests, such as KDM-Age or BloodPhenoAge [124,132]. Of omics-derived BoA, 1H-NMR-based metabolomic aging clocks, such as MetaboAgeMort [44], are currently the most affordable option (i.e., Nightingale Health ∼€34 per sample, yet minimum €10,000 batch requirements apply).

### Potential applications

#### Currently feasible applications

Certain applications are currently possible, yet remain unexplored. Considering some previous studies reported larger effects in those biologically older at baseline [92,93,97,144], BoA may be used as a preliminary screening tool to identify at-risk groups of individuals that may be more likely to respond to a certain intervention a priori. To clarify, this does not imply using BoA to predict response, because other tools designed for this purpose are likely more appropriate and accurate in that aspect, as illustrated by the Personalized Lifestyle Intervention Status (PLIS)-score versus metabolomic aging clocks [145]. Among available markers, we suggest KDM-Age or BloodPhenoAge as the most cost-effective and viable options, because these are largely based on routinely collected clinical data, and can be customized according to the availability of such data using the BioAge package [124]. Both measures reflect physiological dysregulation as compared with the normative reference population, where PhenoAge predicts mortality risk based on physiology [26,124]. A predetermined number of individuals with the highest AgeDev (BA–CA, ideally corrected for CA) would then denote the at-risk group within the study population. Not every marker would be appropriate for such risk assessment, predominantly regarding cost-benefit over conventional measures (e.g., epigenetic clocks likely too expensive), which also remains to be assessed for KDM-Age and BloodPhenoAge.

Furthermore, apart from one [60], all nutritional studies included BoA that reflect systemic aging, providing limited information on underlying mechanisms. It has been proposed that underlying systemic aging includes, among others, different biomarker patterns [37,38] (as described), tissue/organ/system-specific aging rates [29,137], and adaptive or damaging processes [43,146]. To elaborate on the latter, altering adaptation mechanisms that are neutral to or have been designed to protect against deteriorative aging processes may be harmful, whereas reducing damaging processes that accelerate aging could be beneficial [146]. Several mechanistic BoA have been developed that provide insights potentially overlooked by systemic markers, for example, DamAge, AdaptAge [43], and organ aging clocks [128,131,130]. Notably, through Mendelian randomization, DamAge and AdaptAge include CpGs possibly causal to aging [43]. Besides, organ clocks have shown promise in predicting organ-specific health outcomes, aligning with the notion that one is as old as one’s oldest organ [29]. We expect such BoA, and aging patterns, to be of added value besides systemic aging markers, and hypothesize that intra and interindividual heterogeneity to some extent explain marginal effects found in nutrition studies thus far, and obstruct comparison of studies. Essentially individuals with corresponding systemic AgeDev within a population could differ in underlying mechanistic aspects. The addition of causally and mechanistically informed markers in future nutrition studies is not limited to BoA, which requires further testing and in most part remains to be trained on outcomes other than CA, but can comprise alternative informative outcomes, for example, validated conventional markers, data on nutrient status, absorption or other physiological features. Understanding heterogeneity in aging and related impact of nutrition, through the identification of antiaging dietary factors and corresponding mechanisms, could concurrently offer valuable insights into aging biology.

#### Future applications

**FIGURE 3:**
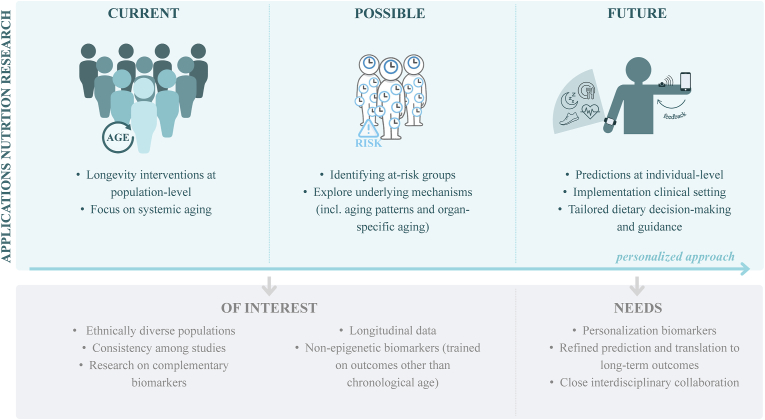
Summary of current, possible, and future applications of biomarkers of aging in nutrition research. “of interest” indicates aspects that could improve current or possible applications and “needs” requirements to enable future applications.

We expect that future applications of BoA will involve personalized approaches, such as individual-level predictions and tailored health-oriented advice. Therein, advances in predictive capacity, mechanistic insights and technology, as well as close interdisciplinary collaboration among nutritionists, biogerontologists and biomarker developers will be essential. Generally, BoA are predictive, and therefore applied on a population level, assessing one-size-fits-all strategies. The effectiveness of certain nutritional strategies is expected to be more profound in specific subgroups or individuals, largely attributed to heterogeneity in underlying processes and preferences [147]. Realizability of reliable individual-level (pace of) aging clocks can be questioned owing to, among others, technical noise and potential personal fluctuations [11,148]. We argue that personalization and further training would be required, for example, incorporation of personal data that reflect individual heterogeneity. Herein, biomarker developers will play a primary role. Aging patterns are a promising first step toward more personalized approaches by identifying subgroups within populations and individual aging pathways [37,38]. Yet, primarily conceptual, rather than exact current measures. Before implementation at the individual level, predictive performance within individuals necessitates further understanding and validation in larger longitudinal studies, also considering use in clinical setting. Future research should consider exploration of novel patterns that are comprehensive yet practical in terms of costs, computational demand and complexity. Eventually, distinct biomarker fingerprints, related to organ and systems aging, may facilitate tailored decision-making regarding nutrition for healthy aging [29]. Although addition of phenotypic data may not enhance compliance with personalized dietary advice [149], it may effectuate larger health improvements. Emerging digital health technologies could prove useful in future personalized nutrition and aging approaches, as described elsewhere [150,151]. Figure 3 summarizes the current and potential applications of BoA in nutrition research. Before proceeding to aspirational future applications, efforts to validate, refine, as well as research the biological underpinning of existing BoA should be prioritized.

## Conclusions and Future Perspectives

BoA are promising, advancing tools for the identification of nutritional strategies and related healthy aging mechanisms. However, several challenges ought to be addressed or considered on the implementation of BoA in nutritional research. We have elaborated on practical implications, current and potential applications, and provided actionable guidance so that nutritional researchers can make informed decisions on contextually appropriate BoA, to improve consistency of implementation. In this last section, we will provide future research directions.

Most importantly, the predictive value and long-term impact of modification of these markers require immediate attention. Additional longitudinal testing and further advancements are warranted for the validation of short(er)-term predictions by BoA. In agreement with prior work [28], we stress the need for large-scale cohorts with long-term follow-up periods of several years, or ideally even decades until incidence of morbidity or mortality, including diverse populations and biomarkers. Nutrition researchers should strive to improve the availability of high-quality nutrition data at the same timepoints as input data for BoA in observational-setting, and set up well-designed studies using relevant BoA as primary endpoint in experimental settings. Exploring the inclusion of mechanistically informed markers or input data other than DNA methylation, such as metabolomics, could provide important insight into aging heterogeneity and dietary influences, and concurrently enhance BoA credibility through understanding of underlying (causal) constructs.

On refinement of BoA, more specific guidelines for standardized use can be formulated. Enhanced consistency and comparability of forthcoming nutritional research may simultaneously contribute to the identification of nutritional antiaging strategies, to prioritize for long-term evaluation [10], and BoA validation. On the basis of previous research, plant-rich diets, IF, FMD or other anti-inflammatory dietary strategies would be interesting to investigate further, with necessary adaptations to ensure suitability and feasibility across diverse populations. Additionally, considering most research has been hypothesis-driven, data-driven approaches may aid the discovery of promising dietary components that have remained largely unexplored. Beyond diet, incorporation of other lifestyle or environmental factors should be considered, and consideration should be given to strategies to improve eventual adherence to dietary guidelines.

To conclude, despite remaining knowledge gaps and much needed advancements that should be taken into consideration on implementation, we consider BoA exiting tools that to different extents show promise in nutrition research by facilitating the identification of potential healthy aging strategies in relatively short durations, advancing insight into the important role of nutrition during aging.

## Author contributions

The authors’ responsibilities were as follows – KNML: wrote the manuscript; PG, GC, LCPGMdG: offered critical and valuable insights; and all authors: read and approved the final manuscript.

## Data availability

Data described in the manuscript, codebook, and analytic code will be made available on request pending application and approval.

## Declaration of generative AI and AI-assisted technologies in the writing process

During the preparation of this work, the author(s) used ChatGTP to improve language. After using this tool/service, the author(s) reviewed and edited the content as needed and take(s) full responsibility for the content of the publication.

## Funding

This research received no external funding.

## Conflict of interest

LCPGMdG is an Editor for Advances in Nutrition and played no role in the Journal's evaluation of the manuscript. The other authors declare that they have no known competing financial interests or personal relationships that could have appeared to influence the work reported in this article.
