# Supplementary material for: Perspective: Biomarkers of Aging in Human Nutrition Research—A Focus on Applications, Challenges, and Opportunities
Source: Adv Nutr. 2025 Jul 28;16(9):100486. doi: 10.1016/j.advnut.2025.100486 (PMC12410545; doi:10.1016/j.advnut.2025.100486)
Supplement: Multimedia component 1 [file mmc1.pdf]

Supplementary Material

Perspective: Biomarkers of Aging in Human Nutrition Research, a focus on applications, challenges and opportunities.

Keeva NM Loughlin*, Pol Grootswagers, Guido Camps and Lisette CPGM de Groot

*Correspondence: Keeva Loughlin, keeva.loughlin@wur.nl

Contents

Supplementary Figures 2

Supplemental Figure 1. Proportions and number of mentions of different epigenetic clocks in Titles, Abstracts and Keywords in the Scopus database over time, sorted by generation. 2

Supplemental Figure 2. Decision flowchart on the selection of relevant biomarkers of aging for nutrition research. 3

Supplemental Material 1: Interpretation of results hypothetical research situations 4

Supplementary Figures

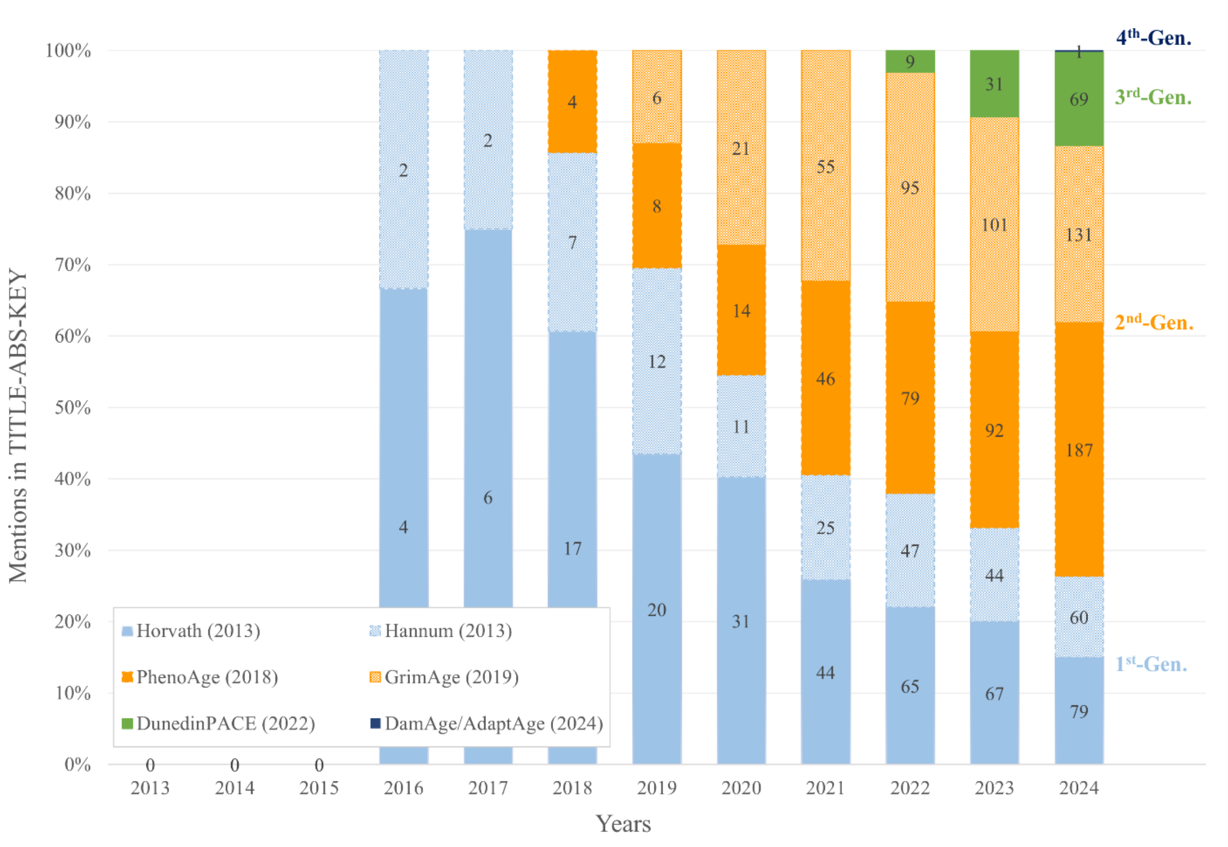

Supplemental Figure 1. Proportions and number of mentions of different epigenetic clocks in Titles, Abstracts and Keywords in the Scopus database over time, sorted by generation.

The y-axis represents the proportion of mentions of the specified epigenetic clock in Titles, Abstracts or Keywords of all document types within Scopus, relative to all mentions of these six epigenetic clocks. The numbers within the bars represent the exact number of documents identified through the search. The following search queries were performed separately: 1) TITLE-ABS-KEY ("HorvathAge*" OR "*HorvathAge" OR (Horvath* W/3 age) OR (Horvath* W/2 clock) OR "HorvathDNAmAge*" OR (Horvath* W/3 DNAm*) OR (Horvath* W/4 *AA) OR "AgeAccelHorvath" OR (Horvath* W/1 AgeAccel*) OR "PCHorvath*" OR (Horvath* W/2 PC*) OR "Horvath Acceleration"); 2) TITLE-ABS-KEY ("HannumAge*" OR "*HannumAge" OR (Hannum* W/3 age) OR (Hannum* W/2 clock) OR "HannumDNAmAge*" OR (Hannum* W/3 DNAm*) OR (Hannum* W/4 *AA) OR (Hannum* W/1 AgeAccel*) OR "PCHannum*" OR (Hannum* W/2 PC*) OR "Hannum Acceleration"); 3) TITLE-ABS-KEY (PhenoAge* OR "Pheno Age*" OR "PCPhenoAge*" OR "DNAmPhenoAge*" OR (Pheno* W/1 AgeAccel*) OR "Pheno-AA" OR "PhenoAA"); 4) TITLE-ABS-KEY (GrimAge* OR "Grim Age*" OR "PCGrimAge*" OR "DNAmGrimAge*" OR (Grim* W/1 AgeAccel*) OR "Grim-AA" OR "GrimAA"); 5) TITLE-ABS-KEY (DunedinPACE* OR "Dunedin PACE*"); 6) TITLE-ABS-KEY ((DamAge* AND AdaptAge*) OR YingAdaptAge* OR YingDamAge*). The search yielded no results between 2013 and 2015. In the legend, the year each epigenetic clock was developed is provided between brackets. The colours indicate the different generations. 1st-Gen. = first-generation clock (light blue); 2nd-Gen. = second-generation clock (orange); 3rd-Gen. = third-generation clock (green); 4th-Gen =fourth-generation clock (dark blue).

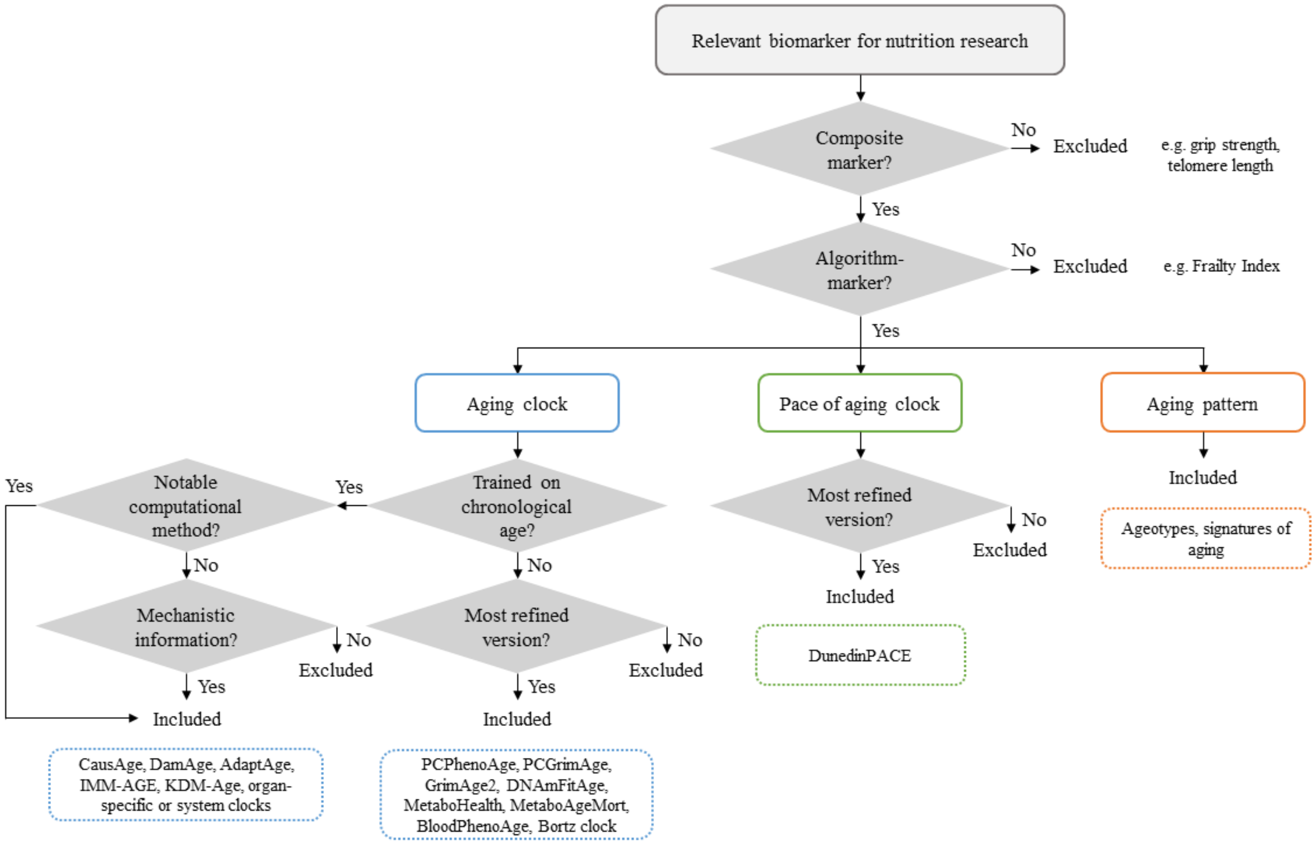

Supplemental Figure 2. Decision flowchart on the selection of relevant biomarkers of aging for nutrition research.

The decision flowchart refers to the final selection of suggested biomarkers of aging currently relevant to nutrition research in Table 4 of the main text. The flowchart has been categorized per type of biomarker of aging: aging clock (blue), pace of aging clock (green), and aging pattern (orange). Note that MetaboHealth is not officially an aging clock because it does not provide biological age as outcome, but rather provides a risk score for mortality.

Supplemental Material 1: Interpretation of results hypothetical research situations

Situation 1: In a general middle-aged population, a 3-month Fasting Mimicking Diet (FMD) significantly reduces the increase in DunedinPACE and DamAge, but does not affect GrimAge2, PCPhenoAge, AdaptAge and CausAge, as compared to a habitual control diet.

First of all, in longitudinal studies both the intervention group and the control group may show an increase in aging measures, due to the passing of time. Decelerative effects of an intervention include both an increase less than the control group or an overall reduction significantly different from the change in the control group. Considering DunedinPACE reflects current aging, while aging clocks reflect past aging until the moment of measurement [1], it can be expected that DunedinPACE responds more rapidly to intervention. An effect in current aging may eventually result in an effect on overall aging, yet the 3-months in this example may have been too short to exert such effects. Note that, cross-sectionally developed biomarkers may be contaminated by non-age-related factors and survival bias, also affecting responsiveness, hence underlying the importance of further longitudinal testing and validation [2]. Moreover, in this example, DamAge was significantly reduced, whereas AdaptAge and CausAge did not change. DamAge reflects damaging changes related to aging, whereas AdaptAge reflects protective changes [3]. Thus, reducing DamAge could be considered beneficial to aging, as can be non-significant changes or (preferably) increases in AdaptAge. DamAge has previously been shown to detect effects of short-term interventions on aging, more so than AdaptAge and CausAge [3]. The effect on CausAge may be diminished because it includes a mixture of both damaging and adaptive sites, and the intervention did not affect AdaptAge [3]. Other aging clocks like GrimAge2 and PCPhenoAge do not distinguish between underlying processes, yet reflect overall systemic aging [4-6]. It is

plausible that an intervention only affects damaging processes, and this concurrently highlights the importance of investigating underlying mechanisms in addition to systemic aging. To conclude, a (hypothetical) 3-month FMD reduced pace of aging and DamAge (i.e. damaging aging processes), did not alter adaptive aging (i.e. protective aging processes), and may have been too short to have an effect on overall systemic aging, as measured by GrimAge2 and PCPhenoAge.

Situation 2: In a young adult population (mean age 25), a 6-month Dietary Approaches to Stop Hypertension (DASH) diet significantly reduced DunedinPACE, PCPhenoAge, but not GrimAge2, BloodPhenoAge, KDM-Age and ECGAge, as compared to a control habitual diet.

Compared to blood-biochemistry-based or clinical phenotype-based markers, such as BloodPhenoAge [6], KDM-Age [7] and ECG-Age [8], molecular markers like epigenetic clocks may be better at capturing aging in pre-symptomatic or preclinical populations that are more homogenous, such as young adult populations [6]. In other words, BloodPhenoAge, KDM-Age and ECG-Age could be less detective of differences in this hypothetical population, and therefore do not measure an effect. Furthermore, in this hypothetical situation, no effect on GrimAge2 was found. The datasets for development of GrimAge and GrimAge2 comprised of middle-aged to older adults, and reflects (risk of) mortality and lifespan [4, 9]. Further, although still associated with important health outcomes in younger individuals, GrimAge2 was found to not align well with chronological age in this age-group (i.e. Mean Absolute Error 11 years) [4]. Therefore, its performance may be less reliable in younger populations, which could explain the lack of effect in the current example (although for applicability the association with health outcomes is more important than accuracy of chronological age prediction). Off note, this deviation could be mitigated using appropriate statistical techniques [4], though that is beyond the scope of the current

example. Overall, it is essential to recognize that BoA are predominantly developed and tested in general “healthy” populations of European ancestry of diverse ages. If population characteristics of a given nutritional study are very specific (e.g. age group, ethnicity, health-status) or not reflected by the populations used in BoA development, generalizability of a particular BoA should be considered. Uncertainty in generalizability calls for careful implementation, and optionally, comparison of BoA performance (e.g. correlation with chronological age) to previous studies. In unique cases, specialized BoA may be required e.g. gestational age. Lastly, regression-based BoA developed in populations with a wide age range may underestimate age in older individuals and overestimate age in younger individuals (dilute effect) [10]. Returning to the hypothetical situation, 6-months was likely too short for an (structural or functional) effect on ECG-Age, which is based on age-related electrocardiogram data [8]. Its responsiveness remains to be assessed, and considering its development in middle-aged to older individuals, its performance in younger adults. All in all, within the context of this example, most measures were unsuitable to measure aging in younger adults to start with. Yet, the 6-month DASH dietary intervention did significantly decelerate DunedinPACE and PCPhenoAge as compared to the control group, which may be interpreted as a favourable and promising outcome, as these anti-aging effects were observed in a (somewhat more homogenous) younger population. This would serve as a strong illustration of the promise that BoA hold within nutrition and aging research (i.e. assessing aging in preclinical populations), particularly once the impact of such changes on long-term health is evidenced.

References

1. Sugden, K., et al., *Association of Pace of Aging Measured by Blood-Based DNA Methylation With Age-Related Cognitive Impairment and Dementia.* Neurology, 2022. **99**(13): p. e1402-e1413.
2. Belsky, D.W., et al., *DunedinPACE, a DNA methylation biomarker of the pace of aging.* Elife, 2022. **11**.
3. Ying, K., et al., *Causality-enriched epigenetic age uncouples damage and adaptation.* Nat Aging, 2024. **4**(2): p. 231-246.
4. Lu, A.T., et al., *DNA methylation GrimAge version 2.* Aging (Albany NY), 2022. **14**(23): p. 9484-9549.
5. Higgins-Chen, A.T., et al., *A computational solution for bolstering reliability of epigenetic clocks: implications for clinical trials and longitudinal tracking.* Nature Aging, 2022. **2**(7): p. 644-661.
6. Levine, M.E., et al., *An epigenetic biomarker of aging for lifespan and healthspan.* Aging (Albany NY), 2018. **10**(4): p. 573-591.
7. Levine, M.E., *Modeling the Rate of Senescence: Can Estimated Biological Age Predict Mortality More Accurately Than Chronological Age?* The Journals of Gerontology: Series A, 2012. **68**(6): p. 667-674.
8. Lima, E.M., et al., *Deep neural network-estimated electrocardiographic age as a mortality predictor.* Nature Communications, 2021. **12**(1): p. 5117.
9. Lu, A.T., et al., *DNA methylation GrimAge strongly predicts lifespan and healthspan.* Aging (Albany NY), 2019. **11**(2): p. 303-327.
10. Clarke, R., et al., *Underestimation of risk associations due to regression dilution in long-term follow-up of prospective studies.* American journal of epidemiology, 1999. **150**(4): p. 341-53.
